# The process of pain assessment in people with dementia living in nursing homes: a scoping review

**DOI:** 10.1177/26323524241308589

**Published:** 2025-01-06

**Authors:** Caroline Kreppen Overen, Maria Larsson, Adelheid Hummelvoll Hillestad, Ingela Karlsson, Siren Eriksen

**Affiliations:** Lovisenberg Diaconal University College, Lovisenberggata 15B, Oslo 0456, Norway Department of Health Sciences, Faculty of Health, Science and Technology, Karlstad University, Karlstad, Sweden; Department of Health Sciences, Faculty of Health, Science and Technology, Karlstad University, Karlstad, Sweden; Lovisenberg Diaconal University College, Oslo, Norway; Department of Health Sciences, Faculty of Health, Science and Technology, Karlstad University, Karlstad, Sweden; Lovisenberg Diaconal University College, Oslo, Norway; The Norwegian National Centre for Ageing and Health, Tønsberg, Norway

**Keywords:** Dementia, nursing, nursing home, pain assessment, palliative care

## Abstract

**Background:**

Pain is a common symptom in people with dementia living in nursing homes, but cognitive impairment, including language and communication difficulties, challenges pain assessment and the ability to self-report pain.

**Objectives:**

This study aimed to identify and summarize patterns, advances, and gaps in research literature describing pain assessment in people with dementia living in nursing homes.

**Design:**

We conducted a scoping review following Arksey and O’Malley’s methodological framework.

**Methods:**

Systematic searches were conducted in CINAHL, Embase, MEDLINE, and PsycINFO. We included studies describing pain expressions in people with dementia and/or healthcare personnel assessment of pain in people with dementia, in a nursing home context. Charted data included demographics, methodological descriptions, ethical and quality assessment and relevant findings. Relevant findings were summarized using thematic analysis, and an overview of patterns, advances, and gaps in the research literature is presented.

**Results:**

Thirty-nine studies were included. The results describe three patterns: (1) pain awareness; (2) suspected pain and (3) pain mapping. Collectively, these patterns constitute a process of pain assessment, integrating pain expressions of people with dementia. Important perspectives on self-reporting are touched upon in several of the included studies, though direct descriptions of attempts to capture the residents’ own experience of pain are sparse.

**Conclusion:**

This scoping review provides a comprehensive description of pain assessment in people with dementia living in nursing homes as a process in three steps. We identified several knowledge gaps in the understanding of this process and provide concrete recommendations for further research. The results underpin the importance of pain assessment approaches that incorporate the flexibility to meet residents’ varying and potentially fluctuating ways of communicating pain.

**Trial registration:**

This scoping review is registered in the Open Science Framework (https://osf.io/8kaf5/).

## Background

The nursing home population is characterized by a high degree of multimorbidity^1^ and polypharmacy^2^ and a large proportion of nursing home residents have a moderate-to-severe degree of dementia.^3,4^ Studies have documented a pain prevalence in people with dementia living in nursing homes of 35%–43%,^2–4^ but a possible prevalence of 60%–80%.^5^ Thus, pain assessment is an important part of care for this population.^6^ The International Association for the Study of Pain defines pain as ‘*an unpleasant sensory and emotional experience associated with, or resembling that associated with, actual or potential tissue damage*’.^7^ Pain is a complex multidimensional phenomenon, influenced by physical, psychological, social, cultural, spiritual and existential factors.^8^ Self-reported information is the most appropriate when assessing pain, as symptom experience is subjective and highly personal.^9^ However, for people with dementia living in nursing homes, self-reporting represents a challenge due to cognitive impairment, including difficulties with language and communication.^10–12^ People with dementia might express pain with different behavioural expressions or signs, such as agitation, apathy, restlessness or wandering.^6,13^

The use of assessment tools can supplement challenging pain assessment and support residents’ limitations in communicating verbally. Numerous observational assessment tools targeting pain in people with dementia have been developed and evaluated^10,14^ and systematic use of standardized observational tools has been recommended.^6,15,16^ However, assessment tools only capture fragments of the overall picture.^9^ The ability of people with dementia to self-report is an individual resource that healthcare personnel (HCP) should engage, promote and support.^17^ At some point in the dementia trajectory, extensive cognitive impairment will make self-reporting so difficult that HCP must depend on for instance observational assessment tools.^18^ Nevertheless, HCP can work purposefully to use valid self-reporting for as long as possible.^19,20^

A scoping review by Pringle et al. exploring the complexity of pain recognition, assessment and treatment for people living in nursing homes, found a need for training and detailed guidelines for appropriate assessment of pain in the nursing home population in general.^21^ However, they did not investigate people with dementia in particular, nor focused on knowledge and tools that emphasize accounting for individual variation and the ability to self-report. A systematic review by Tsai et al. investigated the effectiveness of interventions to improve pain assessment and management in people with dementia.^22^ They found that comprehensive pain models improve nurses’ pain assessment and management. However, none of the included interventions emphasized a structured approach to safeguard individuals’ residual capacity to self-report, and the review was concerned about people with dementia in general and not particularly the nursing home population.^22^ Hence, to the best of our knowledge, no study has reviewed the research literature with a comprehensive perspective on pain assessment in people with dementia living in nursing homes, and how the residents’ expressions of pain are integrated into the clinical practice of HCP. Thus, the aim of this scoping review was to identify and summarize patterns, advances and gaps in research literature describing pain assessment in people with dementia living in nursing homes.

## Methods

The Preferred Reporting Items for Systematic Reviews and Meta-Analyses (PRISMA) extension for Scoping Reviews checklist was used to prepare this manuscript.^23^ The procedure presented in this section is derived and extended from a peer-reviewed protocol.^24^ Two or more of the authors were involved in every step of the process, and methodological decisions were discussed extensively. We utilized the five first stages of Arksey and O’Malley’s methodological framework for scoping reviews, with Levac et al.’s recommendations for each stage: (1) Identifying the research questions; (2) Identifying relevant studies; (3) Study selection; (4) Charting the data and (5) Collating, summarizing and reporting the results.^25,26^ The method was additionally advanced by using the PAGER framework (Pattern, Advances, Gaps, Evidence for Practice and Research Recommendations).^27^

### Stage 1: Identifying the research questions

We searched an overview of pain assessment in people with dementia based on the clinical practice of HCP, and how it integrates pain expressions of people with dementia. To clarify the focus of the scoping review, we developed two research questions to target the broad aim of the review:

RQ1: How is the clinical practice regarding pain assessment in people with dementia living in nursing homes described in the research literature?
RQ2: How are pain expressions of people with dementia living in nursing homes described and included in the clinical practice regarding pain assessment?

### Stage 2: Identifying relevant studies

A systematic search in the CINAHL, Embase, MEDLINE and PsycINFO databases was conducted. No time limit for publication was specified. We formed three main blocks in the search strategy: people with dementia (Population), pain expressions in people with dementia and/or HCP’s assessment of pain (Concept of interest) and nursing homes (Context).^28^ The search strategy combines MeSH terms and synonyms within the respective blocks. When developing the search strategy, we observed that the utilization of the search terms in population and context sufficiently reduced the search results, enabling us to apply broad terms for the concept of interest, preventing the exclusion of relevant studies. The search strategy went through several rounds of revision and quality assurance in collaboration with experienced librarians and the full search strategy is available as Supplemental Material (Additional File 1). The main search was carried out in December 2022 and updated in May 2024. The reference lists of the included studies were manually searched.

### Stage 3: Study selection

Inclusion and exclusion criteria are presented in Table 1.

**Table 1.** Eligibility criteria guiding study selection.

Eligibility criteria	Inclusion criteria	Exclusion criteria
Source	Peer-reviewed journals Published in English, Norwegian, Swedish or Danish	Grey literature
Population	Healthcare personnel (such as registered nurses, assistive personnel and doctors) performing care for people with dementia in nursing homes AND/OR People with a diagnosis of dementia (e.g. patients, service users or residents), including people with a researcher diagnosis of dementia (e.g. use of the Mini-Mental State Examination^29^)	Mixed samples (e.g. mild cognitive impairment/cognitive impairment + dementia) Cognitive impairment not caused by dementia Mixed sample where results about people with dementia are not specifically defined in the results
Context	Nursing home	Mixed context where results about nursing homes are not specifically defined in the results
Concept	Research literature describing: Pain expressions in people with dementia living in nursing homes AND/OR Healthcare personnel’s assessment of pain in people with dementia living in nursing homes	Studies that exclusively focus on development and psychometric testing of assessment tools
Study design	Primary research, all study designs	Editorials, commentaries or letters, discussion papers, opinion papers, literature reviews and nonempirical studies

SE and CKO independently reviewed the first 300 abstracts prior to discussing and reaching consensus on the discrepancies. CKO solely reviewed the remaining abstracts. Rayyan^30^ was used as a tool for team-based screening, and sources that subsequently matched the inclusion criteria were obtained for full-text assessment. If the relevance of a study was unclear from the title and abstract, the full article was reviewed. All full texts were independently assessed for eligibility by two researchers. Several calibration meetings were held during the selection process, and disagreements were discussed until consensus was reached.

### Stage 4: Charting the data

Data from 13 studies, randomly selected among the included, were extracted and reviewed by two researchers (CKO and SE) to determine consistency in the understanding of the studies’ compatibility with the research questions and aim. Data from the remaining studies were charted by CKO alone. The final data-charting form was reviewed and approved by all authors, including demographics, aim and research questions, methodological descriptions and relevant findings. Levac et al. argue the importance of quality assessment in scoping reviews to achieve information on the quality of existing knowledge.^26^ Therefore, all authors made an informal assessment of quality during the full-text review and noted any quality deficiencies. Study quality was then assessed using the Mixed Methods Appraisal Tool (MMAT).^31^ SE and CKO independently assessed 10 studies, and CKO solely assessed the remaining. Reflecting the rationale for quality appraisal in scoping reviews, no studies were excluded based on the appraisals.^26^ The importance of ethical awareness in reviews has been emphasized.^32^ In response, we conducted an ethical mapping inspired by Westerdahl et al.^33^ The ethical mapping considered the description of ethical approval, informed consent, data protection, financial support and conflict of interest.

### Stage 5: Collating, summarizing and reporting the results

In this stage, we prepared an overview and summary of the extracted information, which is presented in the results section. The review includes both quantitative and qualitative data. The quantitative results have been transposed into descriptive phrases, and the descriptive summary is formulated in text. Our results are described and discussed in line with the PAGER framework.^27^ Hence, a descriptive thematic analysis of the key findings, was conducted to identify patterns in the research literature; reading, rereading and coding the data, then generating initial themes, which were reviewed and refined several times.^34^ As a scoping review intends to summarize, not synthesize, the results are presented descriptively on a semantic level, using the same terms as those used in the referenced studies where feasible.^25^

## Results

**Figure 1.**
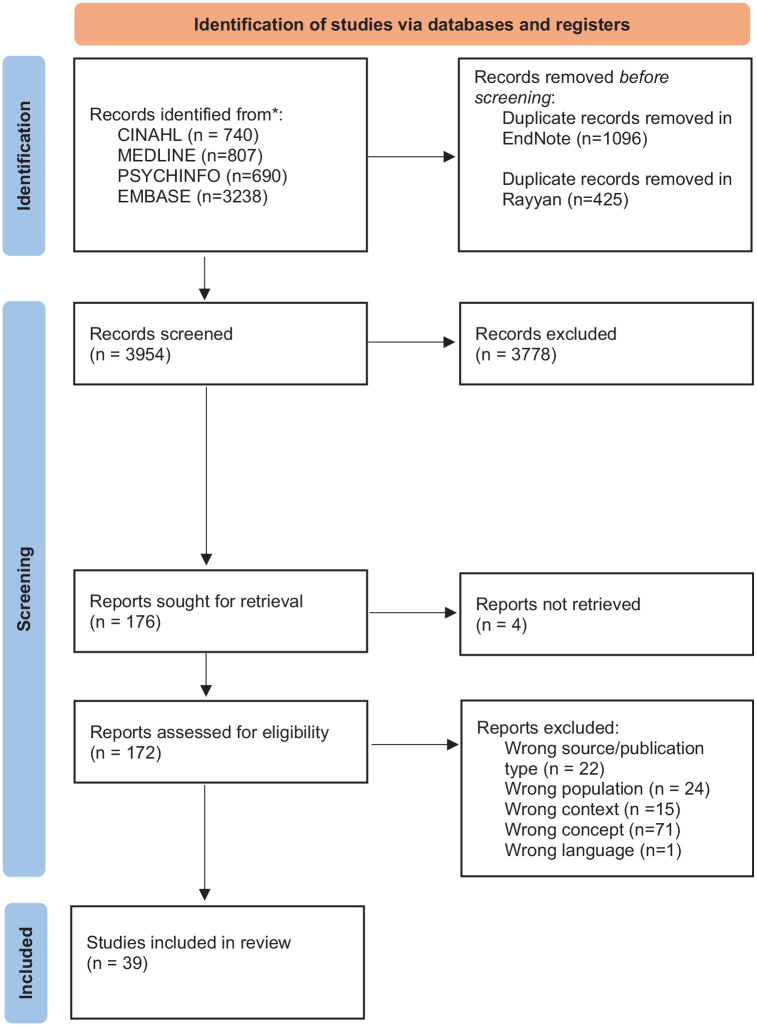
Overview of study selection process. *The updated search (May 2024) identified a total of 446 records from the four databases.

A total of 3954 unique records were assessed by title/abstract after duplicates were removed. The selection process is documented in a PRISMA flowchart (Figure 1).^35^

Thirty-nine studies met all the inclusion criteria. Twenty-three had a quantitative approach, seven a qualitative approach and nine had a mixed or multiple-method approach. The studies were published between 2002 and 2024, in Asia (*n* = 8), Europe (*n* = 9), North America (*n* = 19) and Oceania (*n* = 3). The studies constitute a total sample of 1174 HCP and 37,174 people with dementia. One of the studies included 34,658 people with dementia.^36^ Nursing staff in nursing homes include several different groups of HCP, with substantial international variations in title, level of education and tasks.^37,38^ In our study, we use the collective term HCP, including the diverse array of care providers employed in nursing homes. Where relevant in the presentation of results, we distinguish between registered nurses and assistive personnel, such as certified nursing assistants, nurse assistants and care aides.^37^ Limitations identified with MMAT were mainly related to limited descriptions of methods. In relation to limitations in ethical assessment declaration of adequate data protection was the most common. For an overview of quality- and ethical appraisal, see Supplemental Material (Additional Files 2 and 3). An overview of the included studies is presented in Table 2. An extended version of Table 2, including relevant findings, is available as Supplemental Material (Additional File 4).

**Table 2.** Presentation of studies included.

First author, year, country	Aim/objectives	Participants	Design/method (including assessment tools)
Alexander, 2005, United States^54^	Develop, implement and evaluate a system for pain assessment and monitoring	41 residents with dementia, 24 from secure unit 17 from open unit	Quantitative Pilot study, nonexperimental design *Coloured Visual Analogue Scale (CVAS)*
Andrews, 2019, Australia^43^	Investigate the quality and completeness of pain documentation and assess the extent to healthcare personnel are engaged in documentation processes	114 residents with moderate-to-severe dementia, across 4 facilities. 169 pain episodes	Quantitative Descriptive design Review of medical records
Apinis, 2014, United States^66^	Examine the agreement between the interdisciplinary evaluation and the validated observational pain tools PAINAD and PACSLAC	67 residents with advanced dementia and moderate-to-severe communication disability, from 6 different nursing home wards	Quantitative Cross-sectional *Pain Assessment in Advanced Dementia (PAINAD)* *Pain Assessment Checklist for Seniors with Limited Ability to Communicate (PACSLAC)*
Burns, 2015, United Kingdom^56^	(1) Explore nurses’ knowledge about pain assessment for people with dementia, (2) determine the factors that may influence their knowledge and attitudes towards pain assessment, (3) identify nurses’ level of training and education in pain and dementia, (4) explore the perceived barriers of effective pain assessment	32 registered nurses working in nursing home, regularly caring for people with dementia	Quantitative Cross-sectional survey design Questionnaire, including open-ended questions
Chang, 2011, South Korea^44^	To clarify and conceptualize pain identification in people with dementia by nurses	13 nurses from 3 nursing homes	Quantitative Concept development Individual interviews
Chen, 2015, Taiwan^72^	Investigate the reliability and validity of self-reported pain across groups with different degrees of cognitive function, and to determine the important predictors of self-reported pain intensity in four cognition groups	341 residents diagnosed with dementia from 12 dementia special care units, and 50 registered nurses Control: 73 cognitively intact residents, from 2 long-term care facilities	Quantitative Cross-sectional Multifaceted measures to validate residents’ pain reports *Verbal Descriptor Scale (VDS)* *Doloplus-2*
Chen, 2010, Taiwan^63^	Validate registered nurses’ and nurse assistants’ reports in assessing present pain and to investigate potential influencing factors	304 residents with dementia from 6 dementia special care units 15 registered nurses, 21 nurse assistants	Quantitative Prospective study *Doloplus-2*
Closs, 2003, United Kingdom^65^	(1) Assess the usability of a range of approaches to pain assessments; (2) identify and develop appropriate verbal and/or nonverbal pain assessments in varying levels of cognitive impairment; (3) relate, where possible, the severity of cognitive impairment to the most appropriate methods of assessment	113 nursing home residents	Quantitative Cross-sectional *Verbal Rating Scale (VRS)* *Numerical Rating Scale (NRS)* *Colour Pain Analogue Scale (CS)* *Faces Pain Scale (FS)* *Mechanical Visual Analogue Scale (MVAS)*
Cohen-Mansfield, 2008, United States^64^	Compare pain assessments using self-report, informant rating and observational assessments	153 nursing home residents with dementia from 4 nursing homes 84 staff members	Quantitative Cross-sectional *Functional Pain Scale* *Present Pain Intensity Scale* *Verbal Descriptor Scale* *Global Pain Assessment Scale* *Pain Assessment for Dementing Elderly (PADE)* *Pain Assessment in Noncommunicative Elderly (PAINE)* *Pain Assessment in Advanced Dementia (PAINAD)* *The checklist of nonverbal pain indicators (CNPI)* *Observational Pain Behaviour Assessment Instrument (OPBAI)*
Cohen-Mansfield, 2002, United States^45^	(1) To identify the behaviours and other observable indicators that are perceived by nurses to be manifestations of pain, (2) determine what cues are used to differentiate pain from other causes of unusual behaviour, (3) assess nurses’ perceptions of the prevalence and importance of specific indicators of pain, (4) validate the perceptions of nursing staff members concerning the applicability of the pain indicators provided in the previous studies, (5) to examine their perceptions of their own ability to identify pain in this population	72 staff members from 3 nursing homes	Mixed or multiple methods Individual interviews, survey and focus groups
Cohen-Mansfield, 2002, United States^60^	Examine the reliability and validity of geriatricians’ assessments of pain	79 nursing home residents. 31 with mild/moderate cognitive impairment and 48 with severe cognitive impairment 2 geriatricians	Quantitative Cross-sectional
Corbett, 2016, United Kingdom^40^	Explore the current landscape of pain management in people with dementia living in nursing homes	12 healthcare personnel, including junior care assistants, senior carers, nurses and care home managers	Mixed or multiple methods Triangulation of stakeholder consultation and quality review of pain management Focus groups with care home staff
Ersek, 2011, United States^69^	Explore whether a combination of pain indicators would be significantly better in predicting self-reported pain intensity than any single pain indicator	326 residents, from 24 nursing homes	Quantitative Chart review, resident interviews, surrogate reports from certified nursing assistants *Iowa pain thermometer* *Checklist for nonverbal pain indicators*
Ford, 2015, United States^55^	Examine ethnic differences in the presentation and intensity of nonverbal pain behaviours among African Americans, Caucasians and Hispanics	28 residents with moderate-to-severe dementia and pain-related diagnosis, from 4 nursing homes 6 certified nursing assistants	Quantitative Cross-sectional *Noncommunicative Patients Pain Assessment Instrument (NOPPAIN)*
Gilmore-Bykovskyi, 2013, United States^46^	(1) Examine how nurses make decisions to pharmacologically treat pain, as well as identify the conditions that influence treatment decisions, (2) identify conditions that influence nurses’ actions related to pain management	13 nurses from four facilities (3 licensed practice nurses and 10 registered nurses)	Qualitative In-depth interviews Grounded dimensional analysis
Kaasalainen, 2007, Canada^39^	Explore the decision-making process of pain management of physicians and nurses and how their attitudes and beliefs about pain affect their decisions about prescribing and administering pain medications	24 registered nurses and 33 registered practice nurses from 4 nursing homes 9 physicians	Qualitative Grounded theory Semi-structured, individual interviews
Karlsson, 2012, Sweden^41^	Interpret certified nursing assistants’ perception of pain	12 certified nursing assistants working in dementia care	Qualitative Hermeneutic design Individual interviews
Lautenbacher, 2017, The Netherlands^47^	Identify which facial descriptors are used by caregivers to evaluate and influence their diagnostic decision-making process when assessing pain	284 residents with dementia (mostly advanced stage) from 79 nursing homes	Quantitative Survey Questionnaire
Liu, 2012, China^76^	Report the development and implementation of an observational pain assessment protocol and its impacts on pain management. To report the opinions of the nursing home staff about the protocol	11 healthcare personnel (8 nursing assistants, 2 registered nurses and 1 physiotherapist) 30 residents	Mixed or multiple methods Intervention: Pre-/posttest Group interviews *Chinese version of Pain Assessment in Advanced Dementia* *(C-PAINAD)*
Lundin, 2021, Sweden^48^	Describe the experiences of nurses in caring for people with advanced dementia and pain at the end-of-life	13 registered nurses from 12 nursing homes	Qualitative Descriptive explorative design Individual semi-structured interviews
Manfredi, 2003, United States^57^	(1) Identify a clinical condition consistently described as painful by residents who were able to verbally communicate the experience of pain (2) Assess the reliability and validity of facial expressions as pain indicators in residents with severe dementia undergoing a painful procedure	39 residents with decubitus ulcers able to reliably answer questions about pain 9 residents with dementia and decubitus ulcers	Quantitative
Mezinskis, 2004, United States^49^	Examine which formal and informal methods of pain assessment nurses and caregivers use	From 14 long-term care facilities: Sample A was 160 direct caregivers (35 registered nurses, 41 licensed practice nurses and 84 certified nursing assistants) Sample B was 307 residents in dementia units, with chronic painful illnesses	Quantitative Survey/document analysis Sample A: Questionnaire Sample B: Chart review
Monroe, 2015, United States^50^	Assess nursing home personnel’s cues and practices to identify and alleviate pain	29 healthcare personnel, including registered nurses and licensed practice nurses with direct care responsibilities, from two long-term care facilities	Qualitative Exploratory study Focus group interviews
Monroe, 2014, United States^74^	Determine if a diagnosis of dementia influenced pain self-reports and pain medication use	52 nursing home residents able to self-consent, including 20 people with dementia	Quantitative Between groups, cross-sectional *Discomfort Behaviour Scale*
Monroe, 2012, United States^58^	Use medical records to assess advanced cancer pain at the end-of-life	48 records from 9 nursing homes 43 people with Alzheimer’s dementia (90%), 4 people with vascular dementia (8%) and 1 person with Lewy body dementia (2%)	Quantitative Retrospective between groups cross-sectional design Retrospective chart audit
Nakashima, 2019, United States^36^	Compare pain interventions (including assessment) between nursing home residents with and without dementia	50,673 nursing home residents, 34,658 with dementia	Quantitative Cross-sectional
Neville, 2006, Australia^71^	A needs analysis of the pain management skills of regional nurses caring for older people with dementia	197 staff members (120 unlicensed nurses, 19 enrolled nurses and 55 registered nurses)	Quantitative Survey Questionnaire
Parkman, 2020, United States^51^	(1) Explore the relationship between two observational pain scales, expressed need-driven behaviours and likelihood of medication administration, (2) examined nurses’ perceptions regarding ease of and barriers to use of the scales	28 nursing home residents with dementia 4 registered nurses and 2 licensed practical nurses	Mixed or multiple methods *Abbey Pain Scale* *The Pain Assessment in Advanced Dementia* *(PAINAD)*
Peisah, 2014, Australia^52^	Explore attitudes and processes relating to pain assessment and management	20 staff members (10 registered nurses and 6 nurse assistants)	Quantitative Descriptive design A topical survey typology with semi-structured interviews
Rababa, 2019, Jordan^75^	Examine the relationship among comorbid burden, ability to self-report symptoms, severity of dementia and patient outcomes of pain and agitation	78 nursing home residents with dementia	Quantitative Descriptive correlational design *Discomfort-DAT*
Rababa, 2018, Jordan^70^	Examine temporally based relationships between change in behaviour, the nurses’ level of certainty regarding pain, assessment scope and outcomes of pain	76 nursing home residents with dementia and known pain or a known pain diagnosis	Quantitative Descriptive correlational design *Discomfort-DAT*
Rababa, 2018, Jordan^68^	Examine the associations of pain assessment scope, nurses’ certainty, patient outcomes, and cognitive and verbal characteristics	76 nursing home residents with dementia and known pain/known pain diagnosis	Quantitative Descriptive correlational design *Discomfort-DAT*
Rostad, 2018, Norway^59^	Assess the effectiveness of regular pain assessment on analgesic use and pain score	112 residents with dementia and unable to self-report, from 16 nursing homes that did not routinely use a pain assessment tool	Quantitative Single-blinded, parallel cluster randomized controlled trial *Doloplus-2*
Scherder, 2004, The Netherlands^73^	Compare the assessment by nursing assistants of pain experienced by residents with the residents’ own evaluation	20 residents with Alzheimer’s dementia and 17 residents without dementia, from 2 nursing homes. Both groups with chronic painful conditions	Quantitative Case–control study *Checklist for Nonverbal Pain Indicators (CNPI)* *Coloured Analogue Scale (CAS)*
Sloane, 2007, United States^53^	To describe the amount of staff time spent in care provision of morning care and the sources of discomfort and pain that were identified	17 nursing home residents with dementia who were likely to have chronic pain	Mixed or multiple methods Study and analysis of 51 videotaped morning care and care plans
Vitou, 2022, France^61^	To analyse whether a diagnosis label of Alzheimer’s disease or the stage of the disease may bias pain assessment scores and empathic reactions of healthcare staff in nursing homes	152 certified nursing assistants From 19 nursing homes	Quantitative Experimental between subjects’ design *Visual Analogue Scale (VAS)* *Algoplus*
Vitou, 2021, France^62^	(1) Characterize pain assessment behaviours; (2) compare assessments with individuals with no professional experience in the field of care (controls) and (3) explore the impact of demographic, psychological and socio-professional determinants on pain assessment	50 certified nursing assistants from 5 nursing homes Controls: 96 adults living in the community	Quantitative Experimental between subjects’ design *Visual Analogue Scale (VAS)* *Algoplus*
Yang, 2024, China^42^	To elucidate the methodologies employed by nursing assistants in identification and management of pain	17 nursing assistants	Qualitative Phenomenological design Semi-structured individual interviews
Zahid, 2020, Canada^67^	(1) Evaluate whether pain assessment frequency improved with the use of the tablet app compared with that for the paper-and-pencil method of administration of the PACSLAC-II, (2) evaluate the impact of each method of administration of the PACSLAC-II on frontline staff stress and burnout levels, (3) obtain the perspectives of healthcare personnel on each method of administration	121 staff (33 registered nurses and 88 special care aides)	Mixed or multiple methods Case series design, quasi-experimental and exploratory design *Pain Assessment Checklist for Seniors with Limited Ability to Communicate II* *(PACSLAC-II)*

We identified three patterns in the thematic analysis in which HCP are assessing pain in people with dementia living in nursing homes: (1) pain awareness; (2) suspected pain and; (3) pain mapping. Collectively, these patterns constitute a *process of pain assessment*, which integrate pain expressions of people with dementia. The following presentation of the results is conclusively summarized in an overview of patterns, advances and gaps (Table 4).

### Pattern 1: Pain awareness

HCP must actively search for pain in people with dementia.^39^ ‘Pain awareness’ concerns how HCP are aware that pain might occur, as well as their alertness, knowledge and understanding of the situation. To discover pain, it must be prioritized, and it requires a combination of familiarity with the resident and professional expertise with pain and dementia.^40,41^ Pain awareness can also have a preventive and protective aspect, for example by checking positions to avoid painful pressure ulcers.^41,42^

### Pattern 2: Suspected pain

‘Suspected pain’ refers to the moment when HCP recognize that a person with dementia might be in pain. The included studies describe several sources of suspected/recognized pain: (a) observation of behavioural changes^39–54^; (b) verbal self-reports^43,44,48,53,54^; (c) observation of signs of pain ^41,42,44,45,54^ and (d) known indicators of pain.^44,46,49^ This categorization is based on the conceptual model of how HCP engage in identifying and deciding whether to treat the residents’ pain, developed by Gilmore-Bykovskyi and Bowers.^46^ The model describes how the presence or absence of an obvious reason for pain, influences HCP’s levels of certainty regarding pain. Behavioural change in people with dementia might result in suspected pain but with a high degree of uncertainty – especially in the absence of an obvious reason.^46^ Gilmore-Bykovskyi and Bowers present three groups of behavioural indicators: behaviours suggestive of pain (e.g. repetitive rubbing of a body part), behaviours highly suggestive of pain (e.g. intense guarding with care) and general behaviour changes (e.g. withdrawal or agitation).^46^ Ford et al. compared behavioural pain expressions across different ethnic groups and found no significant differences, only the words used to describe pain.^55^

Observable signs of pain are emphasized, and the most described are (a) behavioural changes that differ from baseline behaviour^42,44–46,49,52,56^; and (b) facial expressions of pain.^41,42,45,47,52–54,57^ ‘Knowing the person’ is highlighted as a crucial prerequisite for recognizing changes from baseline, to identify unique individual pain behaviours and detecting and interpreting pain-related changes in people with dementia.^40–42,44–46,48,52^ Family members are described as important resources,^39,48,56^ as they may be familiar with the residents’ earlier behaviours, and capable of interpreting their present behaviours.^48^ However, though HCP can distinguish behavioural changes from baseline, the behavioural changes might have other causes.^46,50,51^ As Alzheimer’s dementia progresses, observable pain behaviours might diminish and the observation of pain behaviour will be even more difficult.^58^

The different sources of pain identification reported by HCP in the included studies are presented in Table 3.

**Table 3.** Sources of pain recognition reported by healthcare personnel.

Sources of pain recognition^46^	Examples as described in included studies
Observation of behavioural changes	
Unspecified^39,40,43,50–52^	
Behaviours suggestive of pain^40–42,44–50,52^	Grimacing, repetitive rubbing or touching body parts, clenching jaw or fist, bracing body part, changing position, reluctance to move, unusual body movements, moaning, wincing when moved, grunting, whining, sudden limping, tossing and turning in chair or bed, moving head back and forth, body stiffens, sad eyes, dark eyes, empty look, mouth movements, hanging mouth, frowning, narrowed eyes, closed eyes, raising upper lip, opened mouth, tightened lips, empty gaze, seeming disinterested, teary eyed, looking tense, looking sad, looking frightened, curled up position
Behaviours highly suggestive of pain^41,44–46,48–50,52^	Crying, intense guarding, suddenly inability to raise arms, painful look, screams, groaning
General behaviour changes^40–42,44–46,48,49,51^	Withdrawal, restless behaviour, agitation, moodiness, irritability, pacing, sleep disturbance, refusal to eat, depression, unusual quietness, negative vocalizations, decreased participation in activities, changes in sociability, desire to be left alone, anxious behaviour, alterations in daily activities
Resident self-report	
Verbal self-report^43,44,46,48^	Spontaneous self-report, resident response to staff asking about pain
Observation of signs of pain	
Visible signs of pain^41,42,44,45^	Skin colour, oedema in joints, blood on diaper or clothing, changes in vital signs, trembling, falls, limited range of motion, perspiration, contractions
Known indicators of pain	
Visible/obvious reasons for pain^44,45^	Surgery, fracture, terminal
Nonvisible/not obvious reason for pain^44,46,49^	Knowledge of painfull diagnosis, increase in blood pressure

### Pattern 3: Pain mapping

Pain mapping is complex and refers to the specific and more comprehensive part of pain assessment. Pain mapping can be both regulatory-driven (i.e. ‘on admission’) or patient-driven (i.e. ‘the person appears to be in pain’),^52^ where HCP builds upon their suspicion of pain, and/or attempts to determine the underlying cause of the residents stated pain or behaviour that suggests pain. One study found that pain assessment driven by regulation was prevalent.^52^ The state of knowledge is unclear, but there is insufficient evidence to conclude that regular pain mapping using a pain assessment tool is *not* clinically relevant.^59^

There are high validity, reliability and agreement between physicians in the pain assessment of people with dementia with mild/moderate levels of cognitive impairment, but these dropped in the assessment of residents with severe cognitive impairment.^60^ Assistive personnel assigned less pain intensity and affective distress to the person in pain when the person was described as severely ill with Alzheimer’s dementia, compared to when the stage of dementia was not stated.^61^

The perspective of pain mapping in dementia will further be described according to: (a) pain assessment tools; (b) a combination of pain mapping strategies; and (c) self-reporting.

#### Pain assessment tools

Several studies report the use of pain assessment tools as part of pain assessment in clinical practice.^40,53,37,45,46,47,49^ However, the included studies provide limited descriptions of the relationship between the clinical use of assessment tools, degree of dementia and residual capacity to self-report. There are significant differences in HCP use of standardized assessment tools, both interpersonal^62,63^ and between different types of assessment tools.^64^ Registered nurses and assistive personnel using standardized assessment tools largely agreed on the presence of pain at the moment but agreed to a lesser extent on how often pain occurred in the past week.^63^ One study reported poor agreement between tools based on observation compared to self-reports.^64^ Registered nurses reported the use of assessment tools to a greater extent than assistive personnel.^49^

A study by Closs et al found that two-thirds of the participants with moderate or severe dementia were able to use simple self-report assessment scales.^65^ Many of those who when asked, claimed to have no pain indicated that they had pain when they used pain scales.^65^ In contrast, another study found that participants with moderate-to-severe dementia unable to use verbal tools often could use nonverbal tools.^54^

#### Combination of pain mapping strategies

Several of the included studies describe a combination of strategies, where HCP assess and integrate information from various sources including review of medical records^40,50^, physical examination^44,45,66,67^, medical history^44^ and intuition.^48^ The scope of registered nurses’ pain assessment increased with severe dementia and a high degree of uncertainty.^68^ A study investigating the combination and weighting of different sources in pain assessment, found that mapping multiple indicators of pain was not necessarily more appropriate than one single proxy report.^69^ Team meetings with interdisciplinary evaluations of pain for people with dementia report less pain than assessment with standardized observational tools.^66^

Several of the included studies described trialling different combinations of pharmacological and nonpharmacological interventions targeting various potential underlying causes of changed behaviour, including pain.^39,44,46,50,68^ This is described as ‘trial and error’, and the goal is that the person with dementia will return to baseline functioning with the reduction or elimination of their behavioural symptoms.^44,46^

#### Self-reporting of pain

The use of self-reports was highlighted as the most meaningful, when possible.^40^ At the same time, several of the included studies describe the difficulties HCP experience when communicating with people with dementia, and this is one of the major barriers to recognizing and assessing pain in the group.^39,41,42,46,48,51^ There are different points of view when it comes to self-reporting of people with dementia. Two studies stated that a large proportion of the included people with dementia were unable to verbally self-report,^64,70^ and 78% of HCP believed that people with dementia could not accurately provide a self-report of pain,^56^ another study (44%) stated that people with dementia could verbalize at least ‘some pain’ if their pain management were ineffective.^71^ Three of the included studies compared HCP reports of pain with the residents’ reports of pain, and the findings are contradictory.^63,72,73^ People with dementia reported higher prevalence,^63^ intensity and frequency^72^ compared to HCP. On the other hand, assistive personnel is found to score pain as significantly higher than the people with Alzheimer’s dementia themselves.^73^ One study found no significant differences between the prevalence of self-reported pain symptoms when comparing people with and without dementia. People with dementia reported higher pain intensity, were less likely to tell HCP about their pain, and fewer reported that HCP asked about their pain, compared to people without dementia.^74^

Two studies found that a large proportion of the included people with dementia were unable to verbally self-report.^64,70^ Cohen-Mansfield found significantly higher scores on the Mini-Mental State Examination^29^ in the responders to self-report questions, than in non-responders.^64^ Chen and Lin’s findings indicate that people with dementia with up to a moderate level of cognitive impairment may be able to self-report, despite limitations in communication and self-awareness. They highlight that HCP should accept the pain reports of people with dementia to promote adequate pain management, and in addition, use a multifaceted approach for those in the later stages of dementia.^72^

### Integrating the patterns into a coherent process of pain assessment

Collectively, the three identified patterns constitute a pain assessment process.

This process is largely characterized by uncertainty due to cognitive impairment affecting the person’s ability to verbally express pain, and difficulty establishing certainty regarding the underlying causes of pain.^39,46,48,50,51,68,75^ Significantly fewer pain assessments are carried out on people with dementia in nursing homes, compared to people without dementia.^36^

The process of pain assessment involves different HCP disciplines and roles.^39,40,42,52^ To connect the various aspects, the process relies on continuity in relation to communication and information.^39,40,52,60^ Pain assessment is described as a complex network of communication channels in the nursing home, and communication between different disciplines is problematized in several studies.^40–42,52,67^ Poor or inaccurate documentation and communication could be a barrier to effective pain assessment.^51^ Andrews et al. found that 83% of the pain episodes investigated contained documentation only about the problem and the intervention.^43^ The use of a pain management protocol may address these challenges, as it may provide a common language for staff to talk about pain across disciplines and help to strengthen the communication of pain observations.^67,76^ The use of an electronic systematic pain assessment protocol to help HCP identify visual patterns in pain scores over time has been promoted. This could also be a faster and easier way to store and access data.^67^

### Summary of results

We identified three patterns describing the current state and advances of research concerning the pain assessment process in people with dementia living in nursing homes: (1) pain awareness; (2) suspected pain and (3) pain mapping. Patterns, advances and gaps in the research literature concerning pain assessment in people with dementia living in nursing homes are summarized in Table 4.

**Table 4.** Patterns, advances and gaps in the included studies.

Patterns	Advances	Gaps
Healthcare personnel’s clinical practice in pain assessment		
Pain awareness Suspected pain Pain mapping	How uncertainty around pain experience affects pain management processes Observational strategies to detect signs of pain, and the importance of knowledge regarding baseline behaviour HCP perspective on how people with dementia express/self-report pain The importance of continuity in information between shifts and healthcare personnel	Knowledge on • the promotion of systematic individualized pain assessment and how to place the results of assessment tools into a larger context • The application of pain assessment tools in clinical practice (outside the context of participation in studies testing given tools) • how to support people with dementia in communicating their subjective experience of pain • how to assess the residual ability of people with dementia to self-report • how to integrate different pain assessment strategies at different degrees of residual capacity to self-report • how people with dementia experience pain assessment processes in nursing homes • prerequisites for relational continuity in relation to pain assessment • systematic approaches to ensure informational continuity throughout the pain assessment processes • strategies of systematic trial-error where this is unavoidable
(. . .in response to) Pain expressions in people with dementia	Signs of pain (observable, nonverbal) Descriptions of self-reporting focus on the presence and severity of pain Importance of individualized pain assessment	Knowledge on • self-reports of aspects other than presence and severity of pain • cultural differences in pain expressions in people with dementia • the role of relatives in pain assessment

## Discussion

In this review, we aimed to identify and summarize patterns, advances and gaps in research literature describing pain assessment in people with dementia living in nursing homes. We included and examined 39 studies, finding that pain assessment is described as a process, facilitated by uninterrupted information transfer. We identified perspectives of importance on self-reporting, but direct descriptions of self-reporting and attempts to capture the patient’s own experience of pain were sparse.

### Evidence for practice and research recommendations

Our findings highlight and illuminate aspects of pain assessment that are important to reflect on in clinical work with this patient group. Bradbury-Jones states that the evidence for practice using the PAGER framework also targets a broader understanding of the practice field, involving stakeholders beyond clinicians (e.g. researchers).^27^ Evidence to inform practice and research recommendations seen in such a context can contribute by providing concrete recommendations for further research responding to identified knowledge gaps.^27^ The gaps that need to be addressed are presented in Table 4, and the most prominent are elaborated and discussed in this section.

People with dementia’s limited ability to verbally communicate, constitute major challenges and this is highlighted in the literature as a problem that must be addressed.^19^ Hence, the literature is focused on objective assessment alternatives when self-reporting cannot be carried out: these alternatives include the development, testing and implementation of assessment tools.^10^ However, there are nuances between ‘fully capable of self-reporting’ and ‘not at all capable of self-reporting’. Our findings show limited descriptions of how to support people with dementia to communicate their subjective experiences of pain; how HCP can assess the ability/residual ability for self-reporting and how to integrate different pain assessment strategies at different degrees of the residual capacity of the target group to self-report. Self-reporting is mainly described as whether or not the person is able to confirm or deny the presence of pain and to describe the severity of the pain. Descriptions of self-reported pain in the included studies are largely quantified. Qualitative descriptions of the subjective experience of pain are not emphasized, either in those with mild or moderate dementia. Quantitative pain measures are vital in pain management but often overlook important attributes of the subjective experience, such as personal context and meaning, which can have a major impact on the experience of pain.^9^ There is a knowledge gap regarding the promotion of systematic individualized pain assessment and how to place reported pain, the results of assessment tools or clinical examinations into a larger context. Wideman highlights the need for assessment models that specifically emphasize how to address subjectivity related to pain in general.^9^ Our results show that this might be even more challenging in people with dementia. Nevertheless, we claim that models of pain management in this group and context can have the flexibility to meet individual residents’ varying and potentially fluctuating ways of communicating pain, as well as their individual need for assessment, intervention and evaluation.

The results describe ‘trial and error’ strategies: the use of interventions as part of an assessment to find the underlying cause of behavioural changes. Due to risk of delayed treatment, ‘trial and error’ should follow a thorough pain mapping. However, we found that pain mapping will not eliminate all uncertainty, and ‘trial and error’ can be appropriate for instances where uncertainty cannot be eliminated. There is a lack of knowledge concerning strategies for systematic implementation and evaluation of ‘trial and error’, where this is unavoidable. Sandvik et al. discuss how people with dementia receive painkillers as much as or more than people without dementia, in contrast to an earlier trend of undertreating pain due to assessment challenges.^77^ People with dementia in nursing homes constitute a population with a high degree of multimorbidity that is vulnerable to pharmacological side effects.^1,78^ The evaluation of implemented measures is therefore particularly important. These factors highlight the importance of further developing and implementing models that facilitate the systematic evaluation and informational continuity of any pain intervention: both as a result of a specific pain assessment or ‘trial and error’.

We found that pain awareness in particular was described as having a preventive function. Systematic work to prevent pain in this population is described in the included studies to a limited extent. Pain prevention is outside the scope of this review, but in a patient group with such a high prevalence of pain, prevention should be a priority in both clinical practice and future research.^10^ Liao et al. state that there is a lack of knowledge about dementia and pain among HCP, which can be solved with easy access to ongoing training.^79^ Although competence-enhancing measures were outside the scope of this review, we acknowledge this as an important topic that should be highlighted in further studies.

### Strengths and limitations

An important strength of this study was the guidance by a peer-reviewed protocol.^24^ We used an established methodology^25,26^ and analysis method,^34^ as well as standardized reporting guidelines.^23^ To ensure transparency, the review process is described in detail.

This study has some limitations. First, searches, screening and selection of studies are open to error or bias. We acknowledge that this review may not have captured all relevant material, as we did not include grey literature, nor studies published in other languages than English and the Nordic languages. The search strategy resulted in a large volume and wide range of evidence. Another team of researchers might have included and chosen to emphasize other areas of the research field.

We conducted an assessment of quality and ethical standards. Levac et al. argue how quality appraisal is an important aspect of mapping and identifying gaps in the existing literature, giving comprehensive information on the nature and extent of those gaps.^26^ The MMAT guidelines are standardized.^31^ However, the appraisal is vulnerable to bias, as the result depends on the interpretation of the researcher. We sought rigour by involving all members of the research team in the quality appraisal. Studies with low methodological quality are not excluded in this scoping review, following methodological recommendations,^26^ which contributes to a complementary description of the research field. Hence to this, a second limitation is that studies with less robust evidence and a high risk of bias are not excluded, and results must be used cautiously.

## Conclusion

This scoping review provides a comprehensive picture of the existing research on pain assessment in people with dementia living in nursing homes as a process with three steps; it also contributes to the understanding of highly complex nursing processes in this group and context. It has identified several knowledge gaps in the understanding of this process and provides concrete recommendations for further research. The phenomenon of self-reporting in people with dementia is insufficiently explored, and there is limited knowledge on how HCP relates to varying degrees of residual capacity to self-report. The results underpin the importance of pain assessment approaches that have sufficient flexibility to meet individual residents’ varying and potentially fluctuating ways of communicating pain.

## Supplemental Material

sj-docx-1-pcr-10.1177_26323524241308589 – Supplemental material for The process of pain assessment in people with dementia living in nursing homes: a scoping review Supplemental material, sj-docx-1-pcr-10.1177_26323524241308589 for The process of pain assessment in people with dementia living in nursing homes: a scoping review by Caroline Kreppen Overen, Maria Larsson, Adelheid Hummelvoll Hillestad, Ingela Karlsson and Siren Eriksen in Palliative Care and Social Practice

sj-docx-2-pcr-10.1177_26323524241308589 – Supplemental material for The process of pain assessment in people with dementia living in nursing homes: a scoping review Supplemental material, sj-docx-2-pcr-10.1177_26323524241308589 for The process of pain assessment in people with dementia living in nursing homes: a scoping review by Caroline Kreppen Overen, Maria Larsson, Adelheid Hummelvoll Hillestad, Ingela Karlsson and Siren Eriksen in Palliative Care and Social Practice

sj-docx-3-pcr-10.1177_26323524241308589 – Supplemental material for The process of pain assessment in people with dementia living in nursing homes: a scoping review Supplemental material, sj-docx-3-pcr-10.1177_26323524241308589 for The process of pain assessment in people with dementia living in nursing homes: a scoping review by Caroline Kreppen Overen, Maria Larsson, Adelheid Hummelvoll Hillestad, Ingela Karlsson and Siren Eriksen in Palliative Care and Social Practice

sj-docx-4-pcr-10.1177_26323524241308589 – Supplemental material for The process of pain assessment in people with dementia living in nursing homes: a scoping review Supplemental material, sj-docx-4-pcr-10.1177_26323524241308589 for The process of pain assessment in people with dementia living in nursing homes: a scoping review by Caroline Kreppen Overen, Maria Larsson, Adelheid Hummelvoll Hillestad, Ingela Karlsson and Siren Eriksen in Palliative Care and Social Practice

sj-pdf-5-pcr-10.1177_26323524241308589 – Supplemental material for The process of pain assessment in people with dementia living in nursing homes: a scoping review Supplemental material, sj-pdf-5-pcr-10.1177_26323524241308589 for The process of pain assessment in people with dementia living in nursing homes: a scoping review by Caroline Kreppen Overen, Maria Larsson, Adelheid Hummelvoll Hillestad, Ingela Karlsson and Siren Eriksen in Palliative Care and Social Practice

## Appendix

### List of abbreviations

HCP healthcare personnel

MMAT Mixed Methods Appraisal Tool

PAGER Patterns, Advances, Gaps, Evidence for Practice and Research Recommendations

PRISMA Preferred Reporting Items for Systematic Reviews and Meta-Analyses

## Declarations

**Ethics approval and consent to participate:** This study is a scoping review and therefore does not involve collection of primary data. Consequently, formal ethical approval was not required.

**Consent for publication:** Not applicable.

**Availability of data and materials:** The data sets analysed as part of the current study are available from the corresponding author upon reasonable request.
