# Supplementary material for: The process of pain assessment in people with dementia living in nursing homes: a scoping review
Source: Palliat Care Soc Pract. 2025 Jan 6;19:26323524241308589. doi: 10.1177/26323524241308589 (PMC11705334; doi:10.1177/26323524241308589)
Supplement: sj-docx-1-pcr-10.1177_26323524241308589 – Supplemental material for The process of pain assessment in people with dementia living in nursing homes: a scoping review [file sj-docx-1-pcr-10.1177_26323524241308589.docx]

Additional file 1_Search terms

|  | **CINAHL** | **EMBASE** | **MEDLINE** | **PSYCHINFO** |
| --- | --- | --- | --- | --- |
| **Population** | MH («Dementia+») OR MH («Frontotemporal Dementia+») OR MH («Dementia, Multi-Infarct”) OR MH (“Dementia, Presenile+”) OR MH (“Dementia, Senile+”) + MH (“Dementia Patients”) OR MH (“Dementia Vascular+”) OR MH (“Lewy Body disease”) OR MH (“Korsakoff Syndrome”) OR MH (“Alzheimer´s Disease”) OR (TI (dementia) OR AB (dementia)) OR (TI (Alzheimer*) OR AB (Alzheimer*)) OR (TI (Lewy Body) OR AB (Lewy Body)) OR (TI (Korsakoff) OR AB (Korsakoff)) | (exp frontotemporal dementia/ or exp dementia/ or exp multiinfarct dementia/ or exp senile dementia/ or exp presenile dementia/ or exp lewy body/) OR (Exp Wernicke Korsakoff syndrome/) OR ("Alzheimer*".ab,ti.) OR (Korsakoff.ab,ti.) OR (Lewy body.ab,ti.) OR (Dementia.ab,ti.) | (Exp dementia/ or exp alzheimer disease/ or exp dementia, exp vascular/ or exp frontotemporal lobar degeneration/ or exp lewy body disease/) OR (Frontotemporal Dementia/ or Dementia, Multi-Infarct/) OR (Exp Korsakoff Syndrome/) OR (Dementia.ab,ti.) OR ("Alzheimer*".ab,ti.) OR(Lewy body.ab,ti.) OR (korsakoff.ab,ti.) | **(**exp Vascular Dementia/ or exp Dementia/ or exp Presenile Dementia/ or exp Dementia with Lewy Bodies/ or exp Senile Dementia/) OR (exp Alzheimer's Disease/) OR (exp Dementia with Lewy Bodies/) OR (Dementia.ab,ti.) OR (Lewy body.ab,ti.) OR ("Alzheimer*".ab,ti.) OR (Korsakoff.ab,ti.) |
| **Concept of interest** | (MH “Pain+”) OR (MH “Pain measurement”) OR (MH “Pain management”) OR (TI (Pain) OR AB (Pain)) | **(**"Pain".ab,ti.) OR (exp pain/ or exp pain assessment/ or exp pain measurement/) | (exp Pain/ or exp Pain Measurement/ or exp Pain Management/) OR (Pain.ab,ti.) | exp Pain/ or exp Pain Measurement/ or exp Pain Management/) OR ("Pain".ab,ti.) |
| **Context** | (MH «Nursing Homes+») OR (MH Long Term Care) OR (MH Residential care+) OR “nurs* home*” OR “long term care” OR “residential care” OR “care home*” OR “home* for the aged” | **(**exp nursing home/) OR (exp residential care/) OR ("Nurs* home*".af.) OR (Residential care.af.) OR ("care home*".af.) OR (long term care.af.) OR (exp long term care/) | ("Care home*".af.) OR ("Care home*".af.) OR (Residential care.af.) OR ("nurs* home*".af.) OR (exp Residential Facilities/ or exp Nursing Homes/ or exp Homes for the Aged/ or exp Long-Term Care/) | **(**exp Residential Care Institutions/ or exp Nursing Homes/ or exp Long Term Care/) OR ("Nurs* home*".af.) OR ("care home*".af.) OR (residential care.af.) OR ("home* for the aged".af.) OR (long term care.af.) |
