# Supplementary material for: The process of pain assessment in people with dementia living in nursing homes: a scoping review
Source: Palliat Care Soc Pract. 2025 Jan 6;19:26323524241308589. doi: 10.1177/26323524241308589 (PMC11705334; doi:10.1177/26323524241308589)
Supplement: sj-docx-2-pcr-10.1177_26323524241308589 – Supplemental material for The process of pain assessment in people with dementia living in nursing homes: a scoping review [file sj-docx-2-pcr-10.1177_26323524241308589.docx]

| **Author / category of study designs** | **Methodological quality criteria** | **Yes** | **No** | **Can’t tell** | **Comments** |
| --- | --- | --- | --- | --- | --- |
| Alexander, 2005^54^  Quantitative descriptive | 3.1. Are the participants representative of the target population? | x |  |  |  |
|  | 3.2. Are measurements appropriate regarding both the outcome and intervention (or exposure)? | x |  |  |  |
|  | 3.3. Are there complete outcome data? | x |  |  |  |
|  | 3.4. Are the confounders accounted for in the design and analysis? |  |  | x | *No information regarding confounders* |
|  | 3.5. During the study period, is the intervention administered (or exposure occurred) as intended? | x |  |  |  |
| Andrews, 2019^43^  Quantitative descriptive | 4.1. Is the sampling strategy relevant to address the research question? | x |  |  |  |
|  | 4.2. Is the sample representative of the target population? | x |  |  |  |
|  | 4.3. Are the measurements appropriate? | x |  |  |  |
|  | 4.4. Is the risk of nonresponse bias low? | x |  |  |  |
|  | 4.5. Is the statistical analysis appropriate to answer the research question? | x |  |  |  |
| Apinis, 2014^66^  Quantitative descriptive | 4.1. Is the sampling strategy relevant to address the research question? | x |  |  |  |
|  | 4.2. Is the sample representative of the target population? | x |  |  |  |
|  | 4.3. Are the measurements appropriate? | x |  |  |  |
|  | 4.4. Is the risk of nonresponse bias low? | x |  |  |  |
|  | 4.5. Is the statistical analysis appropriate to answer the research question? | x |  |  |  |
| Burns, 2015^56^  Quantitative descriptive | 4.1. Is the sampling strategy relevant to address the research question? | x |  |  |  |
|  | 4.2. Is the sample representative of the target population? | x |  |  |  |
|  | 4.3. Are the measurements appropriate? | x |  |  |  |
|  | 4.4. Is the risk of nonresponse bias low? |  |  | x | *Response rate 33%, no description of non-responders* |
|  | 4.5. Is the statistical analysis appropriate to answer the research question? | x |  |  |  |
| Chang, 2011^44^  Mixed-Methods | 5.1. Is there an adequate rationale for using a mixed methods design to address the research question? | x |  |  |  |
|  | 5.2. Are the different components of the study effectively integrated to answer the research question? | x |  |  | *Limited description* |
|  | 5.3. Are the outputs of the integration of qualitative and quantitative components adequately interpreted? | x |  |  | *Limited description* |
|  | 5.4. Are divergences and inconsistencies between quantitative and qualitative results adequately addressed? |  | x |  |  |
|  | 5.5. Do the different components of the study adhere to the quality criteria of each tradition of the methods involved? |  |  | x |  |
| Chen, 2015^72^  Quantitative non-randomized | 3.1. Are the participants representative of the target population? | x |  |  |  |
|  | 3.2. Are measurements appropriate regarding both the outcome and intervention (or exposure)? | x |  |  |  |
|  | 3.3. Are there complete outcome data? | x |  |  |  |
|  | 3.4. Are the confounders accounted for in the design and analysis? |  |  | x | *Confounders not described* |
|  | 3.5. During the study period, is the intervention administered (or exposure occurred) as intended? | x |  |  |  |
| Chen, 2010^63^  Quantitative descriptive | 4.1. Is the sampling strategy relevant to address the research question? | x |  |  |  |
|  | 4.2. Is the sample representative of the target population? | x |  |  |  |
|  | 4.3. Are the measurements appropriate? | x |  |  |  |
|  | 4.4. Is the risk of nonresponse bias low? | x |  |  |  |
|  | 4.5. Is the statistical analysis appropriate to answer the research question? | x |  |  |  |
| Closs, 2003^65^  Quantitative descriptive | 4.1. Is the sampling strategy relevant to address the research question? | x |  |  |  |
|  | 4.2. Is the sample representative of the target population? | x |  |  |  |
|  | 4.3. Are the measurements appropriate? | x |  |  |  |
|  | 4.4. Is the risk of nonresponse bias low? | x |  |  |  |
|  | 4.5. Is the statistical analysis appropriate to answer the research question? | x |  |  |  |
| Cohen Mansfield, 2008^64^  Quantitative descriptive | 4.1. Is the sampling strategy relevant to address the research question? | x |  |  |  |
|  | 4.2. Is the sample representative of the target population? | x |  |  | *People with dementia in general in aim, but only sampled from NH* |
|  | 4.3. Are the measurements appropriate? | x |  |  |  |
|  | 4.4. Is the risk of nonresponse bias low? |  | x |  | *40% could not use self-reporting* |
|  | 4.5. Is the statistical analysis appropriate to answer the research question? | x |  |  |  |
| Cohen Mansfield, 2002^45^  Mixed methods | 5.1. Is there an adequate rationale for using a mixed methods design to address the research question? | x |  |  |  |
|  | 5.2. Are the different components of the study effectively integrated to answer the research question? | x |  |  |  |
|  | 5.3. Are the outputs of the integration of qualitative and quantitative components adequately interpreted? | x |  |  |  |
|  | 5.4. Are divergences and inconsistencies between quantitative and qualitative results adequately addressed? |  | x |  |  |
|  | 5.5. Do the different components of the study adhere to the quality criteria of each tradition of the methods involved? |  |  | x |  |
| Cohen Mansfield, 2002^60^  Quantitative descriptive | 4.1. Is the sampling strategy relevant to address the research question? | x |  |  |  |
|  | 4.2. Is the sample representative of the target population? | x |  |  |  |
|  | 4.3. Are the measurements appropriate? | x |  |  |  |
|  | 4.4. Is the risk of nonresponse bias low? |  | x |  | *Adapted: complete data on*  *the cases. The medical examination was fully completed for 39,49%* |
|  | 4.5. Is the statistical analysis appropriate to answer the research question? | x |  |  |  |
| Corbett, 2016^40^  Mixed methods | 5.1. Is there an adequate rationale for using a mixed methods design to address the research question? | x |  |  |  |
|  | 5.2. Are the different components of the study effectively integrated to answer the research question? | x |  |  |  |
|  | 5.3. Are the outputs of the integration of qualitative and quantitative components adequately interpreted? |  |  | x |  |
|  | 5.4. Are divergences and inconsistencies between quantitative and qualitative results adequately addressed? |  | x |  |  |
|  | 5.5. Do the different components of the study adhere to the quality criteria of each tradition of the methods involved? | x |  |  |  |
| Ersek, 2011^69^  Quantitative descriptive | 4.1. Is the sampling strategy relevant to address the research question? | x |  |  |  |
|  | 4.2. Is the sample representative of the target population? | x |  |  |  |
|  | 4.3. Are the measurements appropriate? | x |  |  |  |
|  | 4.4. Is the risk of nonresponse bias low? | x |  |  |  |
|  | 4.5. Is the statistical analysis appropriate to answer the research question? | x |  |  |  |
| Ford, 2015^55^  Quantitative descriptive | 4.1. Is the sampling strategy relevant to address the research question? | x |  |  |  |
|  | 4.2. Is the sample representative of the target population? | x |  |  | *Small sample* |
|  | 4.3. Are the measurements appropriate? | x |  |  |  |
|  | 4.4. Is the risk of nonresponse bias low? |  |  | x | *Data from a larger research project, information not available in this study.* |
|  | 4.5. Is the statistical analysis appropriate to answer the research question? | x |  |  |  |
| Gilmore-Bykovskyi, 2013^46^  Qualitative | 1.1. Is the qualitative approach appropriate to answer the research question? | x |  |  |  |
|  | 1.2. Are the qualitative data collection methods adequate to address the research question? | x |  |  |  |
|  | 1.3. Are the findings adequately derived from the data? | x |  |  |  |
|  | 1.4. Is the interpretation of results sufficiently substantiated by data? | x |  |  |  |
|  | 1.5. Is there coherence between qualitative data sources, collection, analysis and interpretation? | x |  |  |  |
| Kaasalainen, 2007^39^  Qualitative | 1.1. Is the qualitative approach appropriate to answer the research question? | x |  |  |  |
|  | 1.2. Are the qualitative data collection methods adequate to address the research question? | x |  |  |  |
|  | 1.3. Are the findings adequately derived from the data? | x |  |  |  |
|  | 1.4. Is the interpretation of results sufficiently substantiated by data? | x |  |  |  |
|  | 1.5. Is there coherence between qualitative data sources, collection, analysis and interpretation? | x |  |  |  |
| Karlsson, 2012^41^  Qualitative | 1.1. Is the qualitative approach appropriate to answer the research question? | x |  |  |  |
|  | 1.2. Are the qualitative data collection methods adequate to address the research question? | x |  |  |  |
|  | 1.3. Are the findings adequately derived from the data? | x |  |  |  |
|  | 1.4. Is the interpretation of results sufficiently substantiated by data? | x |  |  |  |
|  | 1.5. Is there coherence between qualitative data sources, collection, analysis and interpretation? | x |  |  |  |
| Lautenbacher, 2017^47^  Quantitative descriptive | 4.1. Is the sampling strategy relevant to address the research question? | x |  |  |  |
|  | 4.2. Is the sample representative of the target population? | x |  |  |  |
|  | 4.3. Are the measurements appropriate? | x |  |  |  |
|  | 4.4. Is the risk of nonresponse bias low? |  | x |  | *Response rate: 22%. No information available regarding the non-responders.* |
|  | 4.5. Is the statistical analysis appropriate to answer the research question? | x |  |  |  |
| Liu, 2012^76^  Mixed-methods | 5.1. Is there an adequate rationale for using a mixed methods design to address the research question? | x |  |  |  |
|  | 5.2. Are the different components of the study effectively integrated to answer the research question? | x |  |  |  |
|  | 5.3. Are the outputs of the integration of qualitative and quantitative components adequately interpreted? |  |  | x |  |
|  | 5.4. Are divergences and inconsistencies between quantitative and qualitative results adequately addressed? | x |  |  |  |
|  | 5.5. Do the different components of the study adhere to the quality criteria of each tradition of the methods involved? |  |  | x | *Limited descriptions of QUAL-methods* |
| Lundin, 2021^48^  Qualitative | 1.1. Is the qualitative approach appropriate to answer the research question? | x |  |  |  |
|  | 1.2. Are the qualitative data collection methods adequate to address the research question? | x |  |  |  |
|  | 1.3. Are the findings adequately derived from the data? | x |  |  |  |
|  | 1.4. Is the interpretation of results sufficiently substantiated by data? | x |  |  |  |
|  | 1.5. Is there coherence between qualitative data sources, collection, analysis and interpretation? | x |  |  |  |
| Manfredi, 2003^57^  Quantitative non-randomized | 4.1. Is the sampling strategy relevant to address the research question? | x |  |  |  |
|  | 4.2. Is the sample representative of the target population? | x |  |  |  |
|  | 4.3. Are the measurements appropriate? | x |  |  |  |
|  | 4.4. Is the risk of nonresponse bias low? | x |  |  | *Adapted: complete data on*  *the cases.* |
|  | 4.5. Is the statistical analysis appropriate to answer the research question? | x |  |  |  |
| Mezinskis, 2004^49^  Quantitative descriptive | 4.1. Is the sampling strategy relevant to address the research question? | x |  |  |  |
|  | 4.2. Is the sample representative of the target population? | x |  |  |  |
|  | 4.3. Are the measurements appropriate? | x |  |  |  |
|  | 4.4. Is the risk of nonresponse bias low? |  |  | x | *No information regarding response rate or non-responders* |
|  | 4.5. Is the statistical analysis appropriate to answer the research question? | x |  |  |  |
| Monroe, 2015^50^  Qualitative | 1.1. Is the qualitative approach appropriate to answer the research question? | x |  |  |  |
|  | 1.2. Are the qualitative data collection methods adequate to address the research question? | x |  |  |  |
|  | 1.3. Are the findings adequately derived from the data? | x |  |  |  |
|  | 1.4. Is the interpretation of results sufficiently substantiated by data? | x |  |  |  |
|  | 1.5. Is there coherence between qualitative data sources, collection, analysis and interpretation? | x |  |  |  |
| Monroe, 2014^74^  Quantitative descriptive | 4.1. Is the sampling strategy relevant to address the research question? | x |  |  |  |
|  | 4.2. Is the sample representative of the target population? | x |  |  | *Small sample* |
|  | 4.3. Are the measurements appropriate? | x |  |  |  |
|  | 4.4. Is the risk of nonresponse bias low? | x |  |  | *Adapted: complete data on*  *the cases.* |
|  | 4.5. Is the statistical analysis appropriate to answer the research question? | x |  |  |  |
| Monroe, 2012^58^  Quantitative descriptive | 4.1. Is the sampling strategy relevant to address the research question? | x |  |  |  |
|  | 4.2. Is the sample representative of the target population? | x |  |  |  |
|  | 4.3. Are the measurements appropriate? | x |  |  |  |
|  | 4.4. Is the risk of nonresponse bias low? | x |  |  | *Response rate 82% (of 11 nursing homes)* |
|  | 4.5. Is the statistical analysis appropriate to answer the research question? | x |  |  |  |
| Nakashima, 2019^36^  Quantitative descriptive | 4.1. Is the sampling strategy relevant to address the research question? | x |  |  |  |
|  | 4.2. Is the sample representative of the target population? | x |  |  |  |
|  | 4.3. Are the measurements appropriate? | x |  |  |  |
|  | 4.4. Is the risk of nonresponse bias low? | x |  |  |  |
|  | 4.5. Is the statistical analysis appropriate to answer the research question? | x |  |  |  |
| Neville, 2006^71^  Quantitative descriptive | 4.1. Is the sampling strategy relevant to address the research question? | x |  |  |  |
|  | 4.2. Is the sample representative of the target population? | x |  |  | *8 male and 183 female* |
|  | 4.3. Are the measurements appropriate? | x |  |  |  |
|  | 4.4. Is the risk of nonresponse bias low? |  |  | x | *Response rate 47%, only information about non-responders on NH level before questionnaire was sent out (13/21 of NH agreed to participate)* |
|  | 4.5. Is the statistical analysis appropriate to answer the research question? | x |  |  |  |
| Parkman, 2020^51^  Mixed methods | 5.1. Is there an adequate rationale for using a mixed methods design to address the research question? | x |  |  |  |
|  | 5.2. Are the different components of the study effectively integrated to answer the research question? | x |  |  |  |
|  | 5.3. Are the outputs of the integration of qualitative and quantitative components adequately interpreted? | x |  |  |  |
|  | 5.4. Are divergences and inconsistencies between quantitative and qualitative results adequately addressed? | x |  |  |  |
|  | 5.5. Do the different components of the study adhere to the quality criteria of each tradition of the methods involved? | x |  |  |  |
| Peisah, 2014^52^  Quan descriptive | 4.1. Is the sampling strategy relevant to address the research question? | x |  |  |  |
|  | 4.2. Is the sample representative of the target population? | x |  |  |  |
|  | 4.3. Are the measurements appropriate? | x |  |  |  |
|  | 4.4. Is the risk of nonresponse bias low? | x |  |  |  |
|  | 4.5. Is the statistical analysis appropriate to answer the research question? | x |  |  | *Adapted: Topical survey typology, hypothesis driven* |
| Rababa, 2018^70^  Quantitative descriptive | 4.1. Is the sampling strategy relevant to address the research question? | x |  |  |  |
|  | 4.2. Is the sample representative of the target population? | x |  |  |  |
|  | 4.3. Are the measurements appropriate? | x |  |  |  |
|  | 4.4. Is the risk of nonresponse bias low? | x |  |  | *Adapted: complete data on*  *the cases.* |
|  | 4.5. Is the statistical analysis appropriate to answer the research question? | x |  |  |  |
| Rababa, 2018^68^  Quantitative descriptive | 4.1. Is the sampling strategy relevant to address the research question? | x |  |  |  |
|  | 4.2. Is the sample representative of the target population? | x |  |  |  |
|  | 4.3. Are the measurements appropriate? | x |  |  |  |
|  | 4.4. Is the risk of nonresponse bias low? | x |  |  | *Adapted: complete data on*  *the cases.* |
|  | 4.5. Is the statistical analysis appropriate to answer the research question? | x |  |  |  |
| Rababa, 2019^75^  Quantitative descriptive | 4.1. Is the sampling strategy relevant to address the research question? | x |  |  |  |
|  | 4.2. Is the sample representative of the target population? | x |  |  |  |
|  | 4.3. Are the measurements appropriate? | x |  |  |  |
|  | 4.4. Is the risk of nonresponse bias low? | x |  |  |  |
|  | 4.5. Is the statistical analysis appropriate to answer the research question? | x |  |  |  |
| Rostad, 2018^59^  Quantitative randomized controlled trials | 2.1. Is randomization appropriately performed? | x |  |  |  |
|  | 2.2. Are the groups comparable at baseline? | x |  |  |  |
|  | 2.3. Are there complete outcome data? | x |  |  |  |
|  | 2.4. Are outcome assessors blinded to the intervention provided? | x |  |  |  |
|  | 2.5 Did the participants adhere to the assigned intervention? | x |  |  | *The nursing home staff completed 84% (800 out of 960) of the scheduled assessments, with a median 19 assessments per participant (range 12–24).* |
| Scherder, 2004^73^  Quantitative non-randomized | 3.1. Are the participants representative of the target population? |  | x |  | *MMSE score 12-24 in the Alzheimer’s dementia group* |
|  | 3.2. Are measurements appropriate regarding both the outcome and intervention (or exposure)? |  |  | x | *No information if tools are validated for people with dementia.* |
|  | 3.3. Are there complete outcome data? | x |  |  |  |
|  | 3.4. Are the confounders accounted for in the design and analysis? |  | x |  | *In results: Data analyses show that patients with AD experienced considerably less pain than the older people without dementia, both at rest and after walking. Cognitive decline = difficult to express pain, abstract thinking.* |
|  | 3.5. During the study period, is the intervention administered (or exposure occurred) as intended? | x |  |  |  |
| Sloane, 2007^53^  Mixed-methods | 5.1. Is there an adequate rationale for using a mixed methods design to address the research question? | x |  |  |  |
|  | 5.2. Are the different components of the study effectively integrated to answer the research question? | x |  |  |  |
|  | 5.3. Are the outputs of the integration of qualitative and quantitative components adequately interpreted? | x |  |  |  |
|  | 5.4. Are divergences and inconsistencies between quantitative and qualitative results adequately addressed? | x |  |  |  |
|  | 5.5. Do the different components of the study adhere to the quality criteria of each tradition of the methods involved? | x |  |  |  |
| Vitou, 2022^61^  Quantitative non-randomized | 3.1. Are the participants representative of the target population? | x |  |  |  |
|  | 3.2. Are measurements appropriate regarding both the outcome and intervention (or exposure)? | x |  |  | *VAS is not a valid tool for a proxy-report of pain in people with severe dementia in*  *a clinical context (the authors argue why they still use it)* |
|  | 3.3. Are there complete outcome data? | x |  |  |  |
|  | 3.4. Are the confounders accounted for in the design and analysis? | x |  |  |  |
|  | 3.5. During the study period, is the intervention administered (or exposure occurred) as intended? | x |  |  |  |
| Vitou, 2021^62^  Quantitative non-randomized | 3.1. Are the participants representative of the target population? | x |  |  |  |
|  | 3.2. Are measurements appropriate regarding both the outcome and intervention (or exposure)? | x |  |  | *VAS is not a valid tool for a proxy-report of pain in people with severe dementia in*  *a clinical context (the authors argue why they still use it)* |
|  | 3.3. Are there complete outcome data? | x |  |  |  |
|  | 3.4. Are the confounders accounted for in the design and analysis? | x |  |  |  |
|  | 3.5. During the study period, is the intervention administered (or exposure occurred) as intended? | x |  |  |  |
| Yang et al, 2024^42^  Qualitative | 1.1. Is the qualitative approach appropriate to answer the research question? | x |  |  |  |
|  | 1.2. Are the qualitative data collection methods adequate to address the research question? | x |  |  |  |
|  | 1.3. Are the findings adequately derived from the data? | x |  |  |  |
|  | 1.4. Is the interpretation of results sufficiently substantiated by data? | x |  |  |  |
|  | 1.5. Is there coherence between qualitative data sources, collection, analysis and interpretation? | x |  |  |  |
| Zahid, 2020^67^  Mixed methods | 5.1. Is there an adequate rationale for using a mixed methods design to address the research question? | x |  |  |  |
|  | 5.2. Are the different components of the study effectively integrated to answer the research question? | x |  |  |  |
|  | 5.3. Are the outputs of the integration of qualitative and quantitative components adequately interpreted? | x |  |  |  |
|  | 5.4. Are divergences and inconsistencies between quantitative and qualitative results adequately addressed? | x |  |  |  |
|  | 5.5. Do the different components of the study adhere to the quality criteria of each tradition of the methods involved? | x |  |  |  |
