# Supplementary material for: The process of pain assessment in people with dementia living in nursing homes: a scoping review
Source: Palliat Care Soc Pract. 2025 Jan 6;19:26323524241308589. doi: 10.1177/26323524241308589 (PMC11705334; doi:10.1177/26323524241308589)
Supplement: sj-docx-3-pcr-10.1177_26323524241308589 – Supplemental material for The process of pain assessment in people with dementia living in nursing homes: a scoping review [file sj-docx-3-pcr-10.1177_26323524241308589.docx]

| Reference | Ethical quality criteria | Documented | Not documented | Comments |
| --- | --- | --- | --- | --- |
| Alexander, 2005^54^ | Was the study approved by a research ethical committee? |  | x |  |
|  | Was informed consent obtained? |  | x |  |
|  | Were adequate measurements taken to protect personal data? |  | x |  |
|  | Is there a declaration on financial support? |  | x |  |
|  | Is there a declaration on potential conflict of interest? |  | x |  |
| Andrews, 2019^43^ | Was the study approved by a research ethical committee? | x |  |  |
|  | Was informed consent obtained? |  | x |  |
|  | Were adequate measurements taken to protect personal data? |  | x |  |
|  | Is there a declaration on financial support? | x |  |  |
|  | Is there a declaration on potential conflict of interest? |  | x |  |
| Apinis, 2014^66^ | Was the study approved by a research ethical committee? | x |  |  |
|  | Was informed consent obtained? | x |  |  |
|  | Were adequate measurements taken to protect personal data? |  | x |  |
|  | Is there a declaration on financial support? | x |  |  |
|  | Is there a declaration on potential conflict of interest? |  | x |  |
| Burns, 2015^56^ | Was the study approved by a research ethical committee? | x |  |  |
|  | Was informed consent obtained? | x |  |  |
|  | Were adequate measurements taken to protect personal data? |  | x |  |
|  | Is there a declaration on financial support? |  | x |  |
|  | Is there a declaration on potential conflict of interest? | x |  |  |
| Chang, 2011^44^ | Was the study approved by a research ethical committee? | x |  |  |
|  | Was informed consent obtained? | x |  |  |
|  | Were adequate measurements taken to protect personal data? |  | x |  |
|  | Is there a declaration on financial support? | x |  |  |
|  | Is there a declaration on potential conflict of interest? |  | x |  |
| Chen, 2015^72^ | Was the study approved by a research ethical committee? | x |  |  |
|  | Was informed consent obtained? | x |  |  |
|  | Were adequate measurements taken to protect personal data? |  | x |  |
|  | Is there a declaration on financial support? |  | x |  |
|  | Is there a declaration on potential conflict of interest? |  | x |  |
| Chen, 2010^63^ | Was the study approved by a research ethical committee? | x |  |  |
|  | Was informed consent obtained? | x |  |  |
|  | Were adequate measurements taken to protect personal data? |  | x |  |
|  | Is there a declaration on financial support? | x |  |  |
|  | Is there a declaration on potential conflict of interest? |  | x |  |
| Closs, 2003^65^ | Was the study approved by a research ethical committee? |  | x |  |
|  | Was informed consent obtained? | x |  |  |
|  | Were adequate measurements taken to protect personal data? |  | x |  |
|  | Is there a declaration on financial support? |  | x |  |
|  | Is there a declaration on potential conflict of interest? |  | x |  |
| Cohen Mansfield, 2008^64^ | Was the study approved by a research ethical committee? |  | x |  |
|  | Was informed consent obtained? | x |  |  |
|  | Were adequate measurements taken to protect personal data? |  | x |  |
|  | Is there a declaration on financial support? | x |  |  |
|  | Is there a declaration on potential conflict of interest? | x |  |  |
| Cohen Mansfield, 2002^45^ | Was the study approved by a research ethical committee? | x |  |  |
|  | Was informed consent obtained? |  | x |  |
|  | Were adequate measurements taken to protect personal data? |  | x |  |
|  | Is there a declaration on financial support? | x |  |  |
|  | Is there a declaration on potential conflict of interest? |  | x |  |
| Cohen Mansfield, 2002^60^ | Was the study approved by a research ethical committee? | x |  |  |
|  | Was informed consent obtained? | x |  |  |
|  | Were adequate measurements taken to protect personal data? |  | x |  |
|  | Is there a declaration on financial support? | x |  |  |
|  | Is there a declaration on potential conflict of interest? |  | x |  |
| Corbett, 2016^40^ | Was the study approved by a research ethical committee? | x |  |  |
|  | Was informed consent obtained? |  | x |  |
|  | Were adequate measurements taken to protect personal data? |  | x |  |
|  | Is there a declaration on financial support? | x |  |  |
|  | Is there a declaration on potential conflict of interest? | x |  |  |
| Ersek, 2011^69^ | Was the study approved by a research ethical committee? | x |  |  |
|  | Was informed consent obtained? | x |  |  |
|  | Were adequate measurements taken to protect personal data? |  | x |  |
|  | Is there a declaration on financial support? | x |  |  |
|  | Is there a declaration on potential conflict of interest? |  | x |  |
| Ford, 2015^55^ | Was the study approved by a research ethical committee? |  | x | *Secondary analysis of data from primary study* |
|  | Was informed consent obtained? |  | x |  |
|  | Were adequate measurements taken to protect personal data? |  | x |  |
|  | Is there a declaration on financial support? | x |  |  |
|  | Is there a declaration on potential conflict of interest? |  | x |  |
| Gilmore-Bykovskyi, 2013^46^ | Was the study approved by a research ethical committee? | x |  | *Exempt from review* |
|  | Was informed consent obtained? | x |  |  |
|  | Were adequate measurements taken to protect personal data? | x |  |  |
|  | Is there a declaration on financial support? | x |  |  |
|  | Is there a declaration on potential conflict of interest? | x |  |  |
| Kaasalainen, 2007^39^ | Was the study approved by a research ethical committee? | x |  |  |
|  | Was informed consent obtained? | x |  |  |
|  | Were adequate measurements taken to protect personal data? |  | x |  |
|  | Is there a declaration on financial support? | x |  |  |
|  | Is there a declaration on potential conflict of interest? | x |  |  |
| Karlsson, 2012^41^ | Was the study approved by a research ethical committee? | x |  |  |
|  | Was informed consent obtained? | x |  |  |
|  | Were adequate measurements taken to protect personal data? |  | x | *They address it, but not how they did it* |
|  | Is there a declaration on financial support? | x |  |  |
|  | Is there a declaration on potential conflict of interest? | x |  |  |
| Lautenbacher, 2017^47^ | Was the study approved by a research ethical committee? | x |  |  |
|  | Was informed consent obtained? |  | x |  |
|  | Were adequate measurements taken to protect personal data? |  | x |  |
|  | Is there a declaration on financial support? | x |  |  |
|  | Is there a declaration on potential conflict of interest? | x |  |  |
| Liu, 2012^76^ | Was the study approved by a research ethical committee? |  | x |  |
|  | Was informed consent obtained? |  | x |  |
|  | Were adequate measurements taken to protect personal data? |  | x |  |
|  | Is there a declaration on financial support? |  | x |  |
|  | Is there a declaration on potential conflict of interest? | x |  |  |
| Lundin, 2021^48^ | Was the study approved by a research ethical committee? |  | x | *Reasons why there was no need* |
|  | Was informed consent obtained? | x |  |  |
|  | Were adequate measurements taken to protect personal data? | x |  |  |
|  | Is there a declaration on financial support? | x |  |  |
|  | Is there a declaration on potential conflict of interest? | x |  |  |
| Manfredi, 2003^57^ | Was the study approved by a research ethical committee? |  | x |  |
|  | Was informed consent obtained? |  | x |  |
|  | Were adequate measurements taken to protect personal data? |  | x |  |
|  | Is there a declaration on financial support? | x |  |  |
|  | Is there a declaration on potential conflict of interest? |  | x |  |
| Mezinskis, 2004^49^ | Was the study approved by a research ethical committee? |  | x |  |
|  | Was informed consent obtained? |  | x |  |
|  | Were adequate measurements taken to protect personal data? |  | x |  |
|  | Is there a declaration on financial support? |  | x |  |
|  | Is there a declaration on potential conflict of interest? |  | x |  |
| Monroe, 2015^50^ | Was the study approved by a research ethical committee? | x |  |  |
|  | Was informed consent obtained? | x |  |  |
|  | Were adequate measurements taken to protect personal data? |  | x |  |
|  | Is there a declaration on financial support? | x |  |  |
|  | Is there a declaration on potential conflict of interest? |  | x |  |
| Monroe, 2014^74^ | Was the study approved by a research ethical committee? | x |  |  |
|  | Was informed consent obtained? | x |  |  |
|  | Were adequate measurements taken to protect personal data? |  | x |  |
|  | Is there a declaration on financial support? | x |  |  |
|  | Is there a declaration on potential conflict of interest? | x |  |  |
| Monroe, 2012^58^ | Was the study approved by a research ethical committee? |  | x | *Secondary analysis of data from primary study* |
|  | Was informed consent obtained? |  | x |  |
|  | Were adequate measurements taken to protect personal data? |  | x |  |
|  | Is there a declaration on financial support? | x |  |  |
|  | Is there a declaration on potential conflict of interest? | x |  |  |
| Nakashima, 2019^36^ | Was the study approved by a research ethical committee? | x |  |  |
|  | Was informed consent obtained? |  | x | *Unique resident identifiers removed before being given to the researchers* |
|  | Were adequate measurements taken to protect personal data? | x |  |  |
|  | Is there a declaration on financial support? | x |  |  |
|  | Is there a declaration on potential conflict of interest? | x |  |  |
| Neville, 2006^71^ | Was the study approved by a research ethical committee? | x |  |  |
|  | Was informed consent obtained? | x |  |  |
|  | Were adequate measurements taken to protect personal data? | x |  |  |
|  | Is there a declaration on financial support? |  | x |  |
|  | Is there a declaration on potential conflict of interest? | x |  |  |
| Parkman, 2020^51^ | Was the study approved by a research ethical committee? | x |  |  |
|  | Was informed consent obtained? | x |  |  |
|  | Were adequate measurements taken to protect personal data? |  | x |  |
|  | Is there a declaration on financial support? |  | x |  |
|  | Is there a declaration on potential conflict of interest? | x |  |  |
| 29. Peisah, 2014^52^ | Was the study approved by a research ethical committee? | x |  |  |
|  | Was informed consent obtained? |  | x |  |
|  | Were adequate measurements taken to protect personal data? |  | x |  |
|  | Is there a declaration on financial support? |  | x |  |
|  | Is there a declaration on potential conflict of interest? | x |  |  |
| 30. Rababa, 2018^70^ | Was the study approved by a research ethical committee? | x |  |  |
|  | Was informed consent obtained? | x |  |  |
|  | Were adequate measurements taken to protect personal data? | x |  |  |
|  | Is there a declaration on financial support? | x |  |  |
|  | Is there a declaration on potential conflict of interest? | x |  |  |
| 31. Rababa, 2018^68^ | Was the study approved by a research ethical committee? | x |  |  |
|  | Was informed consent obtained? | x |  |  |
|  | Were adequate measurements taken to protect personal data? | x |  |  |
|  | Is there a declaration on financial support? | x |  |  |
|  | Is there a declaration on potential conflict of interest? |  | x |  |
| 32. Rababa, 2019^75^ | Was the study approved by a research ethical committee? | x |  |  |
|  | Was informed consent obtained? | x |  |  |
|  | Were adequate measurements taken to protect personal data? |  | x |  |
|  | Is there a declaration on financial support? | x |  |  |
|  | Is there a declaration on potential conflict of interest? | x |  |  |
| Rostad, 2018^59^ | Was the study approved by a research ethical committee? | x |  |  |
|  | Was informed consent obtained? | x |  |  |
|  | Were adequate measurements taken to protect personal data? |  | x |  |
|  | Is there a declaration on financial support? | x |  |  |
|  | Is there a declaration on potential conflict of interest? | x |  |  |
| Scherder, 2004^73^ | Was the study approved by a research ethical committee? | x |  |  |
|  | Was informed consent obtained? | x |  |  |
|  | Were adequate measurements taken to protect personal data? |  | x |  |
|  | Is there a declaration on financial support? | x |  |  |
|  | Is there a declaration on potential conflict of interest? |  | x |  |
| Sloane, 2007^53^ | Was the study approved by a research ethical committee? | x |  |  |
|  | Was informed consent obtained? | x |  |  |
|  | Were adequate measurements taken to protect personal data? |  | x |  |
|  | Is there a declaration on financial support? | x |  |  |
|  | Is there a declaration on potential conflict of interest? | x |  |  |
| Vitou, 2022^61^ | Was the study approved by a research ethical committee? | x |  |  |
|  | Was informed consent obtained? | x |  |  |
|  | Were adequate measurements taken to protect personal data? |  | x |  |
|  | Is there a declaration on financial support? | x |  |  |
|  | Is there a declaration on potential conflict of interest? | x |  |  |
| Vitou, 2021^62^ | Was the study approved by a research ethical committee? | x |  |  |
|  | Was informed consent obtained? | x |  |  |
|  | Were adequate measurements taken to protect personal data? |  | x |  |
|  | Is there a declaration on financial support? | x |  |  |
|  | Is there a declaration on potential conflict of interest? | x |  |  |
| Yang et al, 2024^42^ | Was the study approved by a research ethical committee? | x |  |  |
|  | Was informed consent obtained? | x |  |  |
|  | Were adequate measurements taken to protect personal data? |  | x |  |
|  | Is there a declaration on financial support? | x |  |  |
|  | Is there a declaration on potential conflict of interest? | x |  |  |
| Zahid, 2020^67^ | Was the study approved by a research ethical committee? | x |  |  |
|  | Was informed consent obtained? | x |  |  |
|  | Were adequate measurements taken to protect personal data? |  | x |  |
|  | Is there a declaration on financial support? | x |  |  |
|  | Is there a declaration on potential conflict of interest? | x |  |  |
