# Supplementary material for: The process of pain assessment in people with dementia living in nursing homes: a scoping review
Source: Palliat Care Soc Pract. 2025 Jan 6;19:26323524241308589. doi: 10.1177/26323524241308589 (PMC11705334; doi:10.1177/26323524241308589)
Supplement: sj-docx-4-pcr-10.1177_26323524241308589 – Supplemental material for The process of pain assessment in people with dementia living in nursing homes: a scoping review [file sj-docx-4-pcr-10.1177_26323524241308589.docx]

Additional file 4_Extended presentation of studies included

| **First author, year, country** | **Aim/Objectives** | **Participants** | **Design/Method**  **(including assessment tools)** | **Relevant findings** |
| --- | --- | --- | --- | --- |
| Alexander, 2005^54^  *USA* | Develop, implement and evaluate a system for pain assessment and monitoring. | 41 residents with dementia, 24 from secure unit  17 from open unit | Quantitative  Pilot study, Nonexperimental design  *Colored visual analog scale (CVAS)* | On the secure unit, typical pain target behaviours consisted of verbalization of pain, facial grimacing, crying out, and limping or gait disturbance. Pain target behaviours on the open unit were primarily documented as verbalization of pain.  The residents with severe cognitive impairment, were largely unable to use a verbal assessment tool. Most of the residents on the open unit, with moderate impairment, were also unable to use the verbal tool. In contrast with the verbal pain assessment tool, the non-verbal tool was used successfully for all residents studied. |
| Andrews, 2019^43^  *Australia* | Investigate the quality and completeness of pain documentation and assess the extent to healthcare personnel are engaged in documentation processes. | 114 residents with moderate to severe dementia, across four facilities. 169 pain episodes. | Quantitative  Descriptive design  Review of medical records | Current pain management documentation does not reflect best practice standards.  Only 16% of entries referred to a resident's verbal report of pain, and 2% indicated that the resident had been explicitly asked about the presence of pain. |
| Apinis, 2014^66^  *USA* | Examine the agreement between the interdisciplinary evaluation and the validated observational pain tools PAINAD and PACSLAC. | 67 residents with advanced dementia and moderate to severe communication disability, from six different nursing home wards | Quantitative  Cross-sectional  *Pain assessment in advanced dementia (PAINAD)*  *Pain Assessment Checklist for Seniors with Limited Ability to Communicate (PACSLAC)* | The results of an interdisciplinary evaluation matched the Pain Assessment in Advanced Dementia-tool in 37 of 59 cases. Pain Assessment in Advanced Dementia reported pain in 8 cases (13.6%) where the interdisciplinary evaluation did not report any pain behaviour. The Pain Assessment Checklist for Seniors with Limited Ability to Communicate-tool detected pain while the interdisciplinary evaluation did not in 16 cases out of 59. |
| Burns, 2015^56^  *UK* | (1) Explore nurses' knowledge about pain assessment for people with dementia, (2) determine the factors that may influence their knowledge and attitudes towards pain assessment, (3) identify nurses' level of training and education in pain and dementia, (4) explore the perceived barriers of effective pain assessment. | 32 registered nurses working in nursing home, regularly caring for people with dementia | Quantitative  Cross-sectional survey design  Questionnaire, including open ended questions | The results indicated an overall good knowledge base in relation to the assessment and management of pain in residents with dementia. The majority of respondents recognized that pain in residents with dementia may be evident through a change in behaviour. There was uncertainty in the use of pain assessment tools. All respondents recognized the importance of physiological indicators of pain when assessing residents with dementia. The majority of nurses identified the importance of what family and caregivers report when assessing pain in dementia. Respondents identified that residents with dementia lacked understanding in relation to their pain management, resulting in refusal of analgesia and increased resident anxiety. The majority of respondents thought that people with dementia could not accurately provide a self-report of pain. |
| Chang, 2011^44^  *South-Korea* | To clarify and conceptualize pain identification in people with dementia by nurses. | 13 nurses from three nursing homes | Quantitative  Concept development  Individual interviews | The result presents a concept analysis of nurses identification of pain in people with dementia in a nursing home, where pain identification constitutes three dimensions with several attributes: schematizing based on stages of dementia and types of dementia, checking, and interconnecting the steps that sequentially follow one another after intervention, and cognitively identifying the origins of pain. |
| Chen, 2015^72^  *Taiwan* | Investigate the reliability and validity of self-reported pain across groups with different degrees of cognitive function, and to determine the important predictors of self-reported pain intensity in four cognition groups. | 341 residents diagnosed with dementia from 12 dementia special care units, and 50 registered nurses  Control: 73 cognitively intact residents, from two long term care facilities | Quantitative  Cross-sectional  Multifaceted measures to validate residents’ pain reports  *Verbal descriptor scale (VDS)*  *Doloplus-2* | Although people with dementia had limitations in both communication and self-awareness, those up to a moderate level of cognitive impairment may be capable of using self-reported measures to quantify their pain experience in a reliable and valid fashion. Additionally, the study provides preliminary evidence of the validity of combining both observational assessment and multiple pain indicators to assess pain in residents with advanced dementia. |
| Chen, 2010^63^  *Taiwan* | Validate Registered nurses’ and nurse assistants’ report in assessing present pain and to investigate potential influencing factors. | 304 residents with dementia from six dementia special care units  15 registered nurses  21 nurse assistants | Quantitative  Prospective study  *Doloplus-2* | Across pain variables, fair to moderate agreement was noted in registered nurses’ assessment, but poor agreement in nursing assistants’ assessment. Comparing among pain variables, both registered nurses and nursing assistants obtained a higher level of agreement in the presence of pain, and a lower level of agreement in the frequency of pain. Both registered nurses and nursing assistants tended to report a lower-level intensity and frequency of pain than residents themselves. The findings support the use of registered nurses as proxy pain informants for older people with dementia in the institution, albeit with caution. |
| Closs, 2003^65^  *UK* | (1) Assess the usability of a range of approaches to pain assessments, (2) identify and develop appropriate verbal and/or non-verbal pain assessments in varying levels of cognitive impairment, (3) relate, where possible, the severity of cognitive impairment to the most appropriate methods of assessment. | 113 nursing home residents | Quantitative  Cross sectional  *Verbal rating scale (VRS)*  *Numerical rating scale (NRS)*  *Color pain analogue scale (CS)*  *Faces pain scale (FS)*  *Mechanical visual analogue scale (MVAS)* | Comparing people with dementia with moderate/severe cognitive impairment with people with no/mild cognitive impairment, the results showed that the moderately impaired group and the mild-/no-impairment group was able to complete roughly four or five scales. Those with severe impairment tended, on average, only to be able to complete one scale (usually the verbal rating scale). After one explanation, 68% had completed the verbal rating scale. After two explanations 73% had completed it, and after three explanations 92% had completed the scale. The same general pattern of increase could be seen of all the scales. The results show a tendency for the verbal rating scale to need fewest explanations, with over two-thirds being able to use it after only one explanation. 78 of the participants denied present pain before using the pain scales. A total of 43 of the 78 had moderate to severe cognitive impairment, and 17 of these indicated that they had pain using one or more of the scales. Mann-Whitney showed no difference between the two groups (moderate/ severe impairment VS no/mild impairment) in terms of this aspect of reporting their pain. |
| Cohen Mansfield, 2008^64^  *USA* | Compare pain assessments using self-report, informant rating, and observational assessments. | 153 nursing home residents with dementia from 4 nursing homes  84 staff members | Quantitative  Cross sectional  *Functional Pain Scale*  *Present Pain Intensity Scale*  *Verbal Descriptor Scale*  *Global Pain Assessment Scale*  *Pain Assessment for Dementing Elderly (PADE)*  *Pain Assessment in non-communicative elderly (PAINE)*  *Pain assessment in advanced dementia (PAINAD)*  *The checklist of nonverbal pain indicators (CNPI)*  *Observational pain behavior assessment instrument (OPBAI)* | The results show strongest correlations among assessments of the same type, that is, among different self-report measures, among different observational measures, between 2 informant rating questionnaires, and among 3 informant rating summary questions. Correlations were particularly poor between direct observation assessments and self-report assessments.  40% of the participants were unable to respond to self-report questionnaires because of cognitive impairment. The results show a significant difference between responders and non-responders on the Mini Mental State Examination-score. Those who responded to self-report questions scored higher (mean 11.2, SD 7.1) than those of non-responders (mean 1.6, SD 2.9). |
| Cohen Mansfield, 2002^45^  *USA* | (1)To identify the behaviors and other observable indicators that are perceived by nurses to be manifestations of pain, (2) Determine what cues are used to differentiate pain from other causes of unusual behavior, (3) assess nurses ‘perceptions of the prevalence and importance of specific indicators of pain, (4) validate the perceptions of nursing staff members concerning the applicability of the pain indicators provided in the previous studies, (5) to examine their perceptions of their own ability to identify pain in this population. | 72 staff members from 3 nursing homes | Mixed or multiple methods  Individual interviews, survey and focus groups | Nursing staff members agreed on a core group of behaviours that they perceived as pain indicators. These include specific physical repetitive movements, vocal repetitive movements, physical signs of pain, and changes in behaviour from the norm for that person. The nursing staff members level of familiarity with the residents was reported to have a significant effect on staff members ability to identify and differentiate pain behaviours from other behaviour. |
| Cohen Mansfield, 2002^60^  *USA* | Examine the reliability and validity of geriatricians’ assessments of pain. | 79 nursing home residents. 31 with mild/moderate cognitive impairment and 48 with severe cognitive impairment  2 geriatricians | Quantitative  Cross sectional | The results show reasonable levels of reliability and validity in the pain assessment of persons with mild/moderate levels of cognitive impairment; the agreement rates between geriatricians who had no contact with one another were high. In contrast, when dealing with severely cognitively impaired residents, the reliability and validity dropped. |
| Corbett, 2016^40^  *UK* | Explore the current landscape of pain management in people with dementia living in nursing homes. | 12 healthcare personnel, including junior care assistants, senior carers, nurses and care home managers | Mixed or multiple methods  Triangulation of stakeholder consultation and quality review of pain management  Focus groups with care home staff. | Six major themes in current pain management in dementia: importance of person-centeredness, current lack of pain awareness in staff, communication as a core element, disparities in staff responsibility and confidence, the need for consistency of care and current lack of staff training. |
| Ersek, 2011^69^  *USA* | Explore whether a combination of pain indicators would be significantly better in predicting self-reported pain intensity than any single pain indicator. | 326 residents, from 24 nursing homes | Quantitative  Chart review, resident interviews, surrogate reports from certified nursing assistants  *Iowa pain thermometer*  *Checklist for nonverbal pain indicators* | The results suggest that assessing multiple indicators of pain, including agitation, depressive symptoms, and number of painful diagnoses, do not perform significantly better than a single measured nursing assistant proxy report. |
| Ford, 2015^55^  *USA* | Examine ethnic differences in the presentation and intensity of nonverbal pain behaviors among African American, Caucasian, and Hispanics. | 28 residents with moderate to severe dementia and pain related diagnosis, from four nursing homes  6 certified nursing assistants | Quantitative  Cross sectional  *Non-Communicative Patients Pain Assessment Instrument (NOPPAIN)* | Significant differences were not noted in overall pain intensity ratings across ethnicities. Of the six nonverbal behaviours included, “pain words’’ was the only behaviour that was significantly different between the ethnic groups. |
| Gilmore-Bykovskyi, 2013^46^  *USA* | (1) Examine how nurses  make decisions to pharmacologically treat pain, as well as to identify the conditions what influence treatment decisions, (2) identify conditions that influence nurses’ actions related to pain management. | 13 nurses from four facilities (3 licensed practice nurses and 10 registered nurses) | Qualitative  In-depth interviews  Grounded Dimensional Analysis | Nurses experienced varying levels of certainty regarding suspected pain in response to resident characteristic and whether pain was perceived as visible/obvious or nonvisible/not obvious. Nurses felt highly uncertain about pain in resident with dementia. Suspected pain in resident with dementia was nearly always conceptualized as a change in behaviour to which nurses responded by trialling multiple interventions in attempts to return the resident to baseline, which despite current recommendation, did not include pain relief trials. Residents with dementia were described as at great risk for experiencing underassessment, undertreatment, and delayed treatment for pain. |
| Kaasalainen, 2007^39^  *Canada* | Explore the decision-making process of pain management of physicians and nurses and how their attitudes and beliefs about pain affect their decisions about prescribing and administering pain medications. | 24 registered nurses and 33 registered practice nurses from 4 nursing homes  9 physicians | Qualitative  Grounded theory  Semi structured, individual interviews | Based on grounded theory, a model was developed that highlighted critical decision points for nurses and physicians regarding pain management. The major themes that emerged from the data concerned pain assessment (lack of recognition of pain, uncertainty about the accuracy of pain assessment and diagnosis) and treatment (reluctance to use opioids, working with individualize pain treatments, issues relating to physicians’ trust of the nurse on prescribing patterns). |
| Karlsson, 2012^41^  *Sweden* | Interpret certified nursing assistants’ perception of pain. | 12 certified nursing assistants working in dementia care | Qualitative  Hermeneutic design  Individual interviews | Findings from the study are presented in three themes, with each theme on a level of abstraction, illuminating certified nursing assistants’ perception of pain in people with dementia. Being in a facing phase means being alert to the fact that a person is showing signs of pain or discomfort by perceive facial expressions as well as physical and behavioural signs. Being in a reflective phase means having the ability to reflect, both alone and together with colleagues, supporting one’s perceptions. Being in an acting phase means being receptive to and actively investigating whether the perceived expression is really caused by pain and in relation to this to care preventively and protectively to avoid pain occurring. |
| Lautenbacher, 2017^47^  *The Netherlands* | Identify which facial descriptors are used by caregivers to evaluate and influence their diagnostic decision-making process when assessing pain. | 284 residents with dementia (mostly advanced stage) from 79 nursing homes | Quantitative  Survey  Questionnaire | The study finds which features of facial expressions caregivers rely on when inferring the presence and intensity of pain in people with dementia. Observations of facial expression were most often done during washing, transfer, and mobilization. Some facial descriptor items were used in more than half of all the observations: “frowning” (60%), “narrowed eyes” (51%),“looking tense” (70%), and “looking frightened” (54%); others were used less frequently: “closed eyes” (31%),“raising upper lip” (30%), “opened mouth” (48%), “tightened lips” (45%), “empty gaze” (39%), “seeming disinterested” (37%), “pale face” (37%), “teary eyed” (23%), and “looking sad” (46%). |
| Liu, 2012^76^  *China* | Report the development and implementation of an Observational Pain Assessment Protocol and its impacts on pain management. To report the opinions of the nursing home staff about the protocol. | 11 healthcare personnel (8 nursing assistants, 2 registered nurses and 1 physiotherapist)  30 residents | Mixed or multiple methods  Intervention - Pre-/posttest  Group interviews  *Chinese version of pain assessment in advanced dementia*  *(C-PAINAD)* | Findings from interview (about the use of a pain assessment protocol): The nursing assistants, who were mainly responsible for behavioural pain assessment, stated that the protocol made them more sensitive and responsive to residents’ pain-related behaviour. The protocol encouraged them to undertake pain management in a more systemic manner. It encouraged further comparison of pain levels before and after the pain-relieving interventions. It encourages a standardized and consistent approach in pain assessment and management. It helped to strengthen the communication regarding the outcomes of pain observation and management across various posts in the nursing home. |
| Lundin, 2021^48^  *Sweden* | Describe the experiences of nurses in caring for people with advanced dementia and pain at the end of life. | 13 registered nurses from 12 nursing homes | Qualitative  Descriptive explorative design  Individual semi-structured interviews. | The nurses described communicative, relational, and organizational challenges. One major issue involved difficulties communicating with the person with advanced dementia, resulting in uncertain pain assessment. Other difficulties involved the differentiation of pain from anxiety. Relatives can affect the assessment and management of pain because of their ability to interpret pain behaviour. Pain management was facilitated by good communication with healthcare staff and relatives, extensive professional nursing experience, and already knowing the person with advanced dementia. |
| Manfredi, 2003^57^  *USA* | (1) Identify a clinical condition consistently described as painful by residents who were able to verbally communicate the experience of pain.  (2) Assess the reliability and validity of facial expressions as pain indicators in residents with severe dementia undergoing a painful procedure. | 39 residents with decubitus ulcers able to reliably answer questions about pain  9 residents with dementia and decubitus ulcers | Quantitative | The results suggest that facial expressions, even when viewed in isolation from the clinical context, are valid and reliable indicators of the presence of pain. Clinicians relying on the close observation of facial expressions of patients with severe dementia to infer the presence or absence of pain can expect to be correct between 80% and 90% of the time. When facial expressions are integrated within the clinical context of a painful procedure or disease process, the ability of the clinician to detect the presence of pain is likely to be even higher. |
| Mezinskis, 2004^49^  *USA* | Examine which formal and informal methods of pain assessment nurses and caregivers use. | From 14 long term care facilities:  Sample A was 160 direct caregivers (35 registered nurses, 41 licensed practice nurses, and 84 certified nursing assistants)  Sample B was 307 residents in dementia units, with chronic painful illnesses | Quantitative  Survey/Document analysis  Sample A: Questionnaire  Sample B: Chart review | Registered nurses (68.5%) most often reported the use of assessment tools, followed by some certified nursing assistants (8.3%). Direct caregivers identified informal pain assessment methods that they used; ‘becomes aggressive’, “becomes loud/noisy” and “becomes quiet”. Through logistic regressions of each informal pain assessment method on months of employment or number of residents cared for, no pain assessment method was significantly predicted by these variables. |
| Monroe, 2015^50^  *USA* | Assess nursing home personnel's cues and practices to identify and alleviate pain. | 29 healthcare personnel, including registered nurses and licensed practice nurses with direct care responsibilities, from two long term care facilities | Qualitative  Exploratory study  Focus group interviews | Nurses use the construct of “comfort” and “quality of life” as key components in their overall pain assessment strategy in people with dementia. The process they use involve frequent reassessment and application of interventions geared towards “appearance of comfort”. Nurses reported difficulty in ascertaining whether a person with dements was in pain, and they expressed further difficulty determining the intensity associated with resident pains. Nurses further reported that resident with dementia who are not well known by the staff were greater risk of poor pain management. Nurses had to focus not only on the residents’ comfort, but also the family’s level of comfort with pain management, especially at the end-of-life. |
| Monroe, 2014^74^  *USA* | Determine if a diagnosis of dementia influenced pain self-reports and pain medication use. | 52 nursing home residents able to self-consent, including 20 people with dementia | Quantitative  Between groups, cross-sectional  *Discomfort behavior scale* | Although each group had similar pain-related diagnosis, residents without a dementia diagnosis were significantly more likely to have a medication order for an opioid. Among residents who reported current pain, those with a dementia diagnosis reported greater pain intensity than those without dementia. |
| Monroe, 2012^58^  *USA* | Use medical records to assess advanced cancer pain at the end-of-life. | 48 records from 9 nursing homes  43 people with Alzheimer’s dementia (90%), 4 people with vascular dementia (8%), and 1 person with Lewy Body dementia (2%) | Quantitative  Retrospective between groups cross-sectional design  Retrospective chart audit | Alzheimer’s disease severity was negatively associated with pain behaviours. Post hoc procedures showed that this difference was due to the difference in pain behaviours between individuals with moderate to severe Alzheimer’s disease. As Alzheimer’s disease worsens, observable pain behaviours appear to diminish. |
| Nakashima, 2019^36^  *USA* | Compare pain interventions (including assessment) between nursing home residents with and without dementia. | 50 673 nursing home residents, 34 658 with dementia | Quantitative  Cross-sectional | Residents with dementia had significantly fewer pain assessments than those without dementia. |
| Neville, 2006^71^  *Australia* | A needs analysis of the pain management skills of regional nurses caring for older people with dementia. | 197 staff members (120 unlicensed nurses, 19 enrolled nurses and 55 registered nurses) | Quantitative  Survey  Questionnaire | The analysis indicated that nurses might not have the knowledge base to manage pain effectively. The respondents had essentially negative perceptions of the availability and appropriateness of current pain management education programs. 31% of participants did not know whether there was a pain management resource person in their facility concerning people with dementia.  Many participants did not answer the question asking whether older people with dementia experience pain, reasons being they did not know or felt unqualified to answer the question. The majority of those that did respond (28%) believed that between 91% and 100% of older people with dementia do experience pain; and the majority (44%) also believed that older people with dementia will verbalise at least ‘some pain’ to indicate their pain management is ineffective.  There was generally agreement amongst the participants regarding their clinical practice, except that unlicenced nurses were less likely to agree with people with dementias statement about their pain than Registered Nurses, and unlicenced nurses were also less likely to agree with their colleagues that people with dementia experienced pain. |
| Parkman, 2020^51^  *USA* | (1) Explore the relationship between two observational pain scales, expressed need-driven behaviors, and likelihood of medication administration, (2) examined nurses’ perceptions regarding ease of and barriers to use of the scales. | 28 nursing home residents with dementia  4 registered nurses and 2 licensed practical nurses | Mixed or multiple methods  *Abbey Pain Scale*  *The pain assessment in advanced dementia*  *(PAINAD)* | Qualitative analysis identified three core themes: (a) challenges in assessing people with dementia for pain; (b) facilitators and barriers to pain management, and (c) difficulty caring for people with dementia. |
| Peisah, 2014^52^  *Australia* | Explore attitudes and processes relating to pain assessment and management. | 20 staff members (10 registered nurses and 6 nurse assistants) | Quantitative  Descriptive design  A topical survey typology with semi-structured interviews | Pain assessment and use of pain chart is often regulatory-driven rather than patient-driven. Identification of pain and need for pain relief was ill defined and poorly understood. Both pharmacological and non-pharmacological regimes was used, but in an ad hoc, variable, and unsystematic manner, with patient, staff and attitudinal obstacles between the experience of pain and its relief. Pain charts were not used to monitor the efficacy of pain management.  To recognize pain staff looked for “calling out in pain”, “facial expressions”, “grimacing”, “wincing”, “moaning”, and “frowning”. Behaviour changes was reported as a trigger for pain assessment, usually unspecified “behaviour changes”. Familiarity with residents was reported to guide staff to pick up a distinct indicator with each resident. |
| Rababa, 2019^75^  *Jordan* | Examine the relationship among comorbid burden, ability to self-report symptoms, severity of dementia, and patient outcomes of pain and agitation. | 78 nursing home residents with dementia | Quantitative  Descriptive correlational design  *Discomfort-DAT* | There are significant associations between the severity of dementia and the ability to self-report pain in nursing home residents and nurses’ certainty to pain. Pain assessment scope does not mediate the relationship between nurses’ certainty and patient outcomes.  Nurses were certain about the presence of pain in 26.9% of 78 nursing home residents with dementia prior to assessment. As dementia gets more severe, the nurses become less certain about the presence of suspected pain. Nurses were less certain about the presence of suspected pain (p = 0.024) in nursing home residents with severe dementia compared with that on those with mild dementia. Nurses had low levels of certainty about the presence of suspected pain in NH residents with severe dementia compared with those with moderate dementia (p = 0.027). Nurses were less certain about the presence of pain in nursing home residents with dementia who were unable to self-report pain.  About 20% of the included people with dementia were unable to verbally report pain symptoms. |
| Rababa, 2018^70^  *Jordan* | Examine temporally based relationships between change in behavior, the nurses’ level of certainty regarding pain, assessment scope, and outcomes of pain. | 76 nursing home residents with dementia and known pain or a known pain diagnosis | Quantitative  Descriptive correlational design  *Discomfort-DAT* | Comorbid burden and ability to verbally self-report symptoms were found to be significantly predictors of patient outcomes of pain and agitation. However, the ability to verbally self-report did not explain the relationship between comorbid burden and patient outcomes.  The majority of residents in the current study were unable to verbally self-report symptoms |
| Rababa, 2018^68^  *Jordan* | Examine the associations of pain assessment scope, nurses’ certainty, patient outcomes, and cognitive and verbal characteristics. | 76 nursing home residents with dementia and known pain/known pain diagnosis | Quantitative  Descriptive correlational design  *Discomfort-DAT* | The three hypotheses were supported:   1. Certainty of suspected pain by the nurse will be associated with the scope of pain assessment provided to a person with dementia who has a change in condition 2. Pre-assessment level of nurses’ certainty, assessment scop, and post-assessment level of certainty will be associated with pain outcome 3. Post-assessment certainty is a unique significant predictor of resident outcome |
| Rostad, 2018^59^  *Norway* | Assess the effectiveness of regular pain assessment on analgesic use and pain score. | 112 residents with dementia and unable to self-report, from 16 nursing homes that did not routinely use a pain assessment tool | Quantitative  Single-blinded, parallel cluster randomized controlled trial.  *Doloplus-2* | The study failed to document effectiveness of regular pain assessment on pain score or analgesic use in the sample. However, there is not sufficient evidence to conclude that regular pain assessment using a pain assessment tool is not clinically relevant. |
| Scherder, 2004^73^  *The Netherlands* | Compare the assessment by Nursing Assistants of pain experienced by residents with the residents’ own evaluation. | 20 residents with Alzheimer dementia and 17 residents without dementia, from 2 nursing homes. Both groups with chronic painful conditions. | Quantitative  Case–control study  *Checklist for non-verbal pain indicators (CNPI)*  *Colored analog scale (CAS)* | Nurse assistants’ pain scores were significantly higher than those of the patients with Alzheimer’s dementia, both at rest and after walking. |
| Sloane, 2007^53^  *USA* | To describe the amount of staff time spent in care provision of morning care and the sources of discomfort and pain that were identified. | 17 nursing home residents with dementia who were likely to have chronic pain | Mixed or multiple methods  Study and analysis of 51 videotaped morning care and care plans. | The most common methods of communicating pain and discomfort were verbal statements (88% of participants), facial grimacing (71%), stiffened posture or other guarding behaviours (59%), rubbing a body part (52%), gripping tightly to the caregiver (53%), and increased irritability or lability (52%). |
| Vitou, 2022^61^  *France* | To analyze whether a diagnosis label of Alzheimer’s disease or the stage of the disease may bias pain assessment scores and empathic reactions of health care staff in nursing homes. | 152 certified nursing assistants  From 19 nursing homes | Quantitative  Experimental between subjects’ design  *Visual analogue scale (VAS)*  *Algoplus* | Alzheimer’s disease label had no influence on pain assessment scores. The stage of the disease had a significant effect on the health care staff assessments with severe stage associated with lower pain intensity scores and empathic reactions. |
| Vitou, 2021^62^  *France* | (1) characterize pain assessment behaviors (2) compare assessments with individuals with no professional experience in the field of care (controls); and (3) explore the impact of demographic, psychological, and socio-professional determinants on pain assessment. | 50 certified nursing assistants from 5 nursing homes  Controls: 96 adults living in the community | Quantitative  Experimental between subjects’ design  *Visual analogue scale (VAS)*  *Algoplus* | The results highlighted the interrater variability of pain assessments in people with dementia in certified nursing assistants and control participants, even with standardized observational tools. |
| Yang et al, 2024^42^  *China* | To elucidate the methodologies employed by nursing assistants in identification and management of pain. | 17 nursing assistants | Qualitative  Phenomenological design  Semi-structured individual interviews | The results describe four themes: 1) perception of pain; 2) strategies for coping with pain; 3) emotional and psychological responses to pain-related caregiving; and 4) challenges and needs in pain-related caregiving. |
| Zahid, 2020^67^  *Canada* | (1) Evaluate whether pain assessment frequency improved with the use of the tablet app compared with that for the paper-and-pencil method of administration of the PACSLAC-II, (2) evaluate the impact of each method of administration of the PACSLAC-II on frontline staff stress and burnout levels, (3) obtain the perspectives of healthcare personnel on each method of administration. | 121 staff (33 registered nurses and 88 special care aides) | Mixed or multiple methods  Case series design, quasi-experimental and exploratory design  *Pain Assessment Checklist for Seniors with Limited Ability to Communicate II*  *(PACSLAC-II)* | The results show that the use a tablet-app version of the “Pain Assessment Checklist for Seniors with limited ability to communicate” provide easy access to identify visual patterns in pain scores over time using a graph instead of number. Generally, it provides a common language for staff across disciplines to talk about pain. The primary barrier associated with changes in pain assessment practices identified by frontline staff was miscommunication across disciplines. |
